# Gut bacteria identified in colorectal cancer patients promote tumourigenesis via butyrate secretion

**DOI:** 10.1038/s41467-021-25965-x

**Published:** 2021-09-28

**Authors:** Shintaro Okumura, Yusuke Konishi, Megumi Narukawa, Yuki Sugiura, Shin Yoshimoto, Yuriko Arai, Shintaro Sato, Yasuo Yoshida, Shunya Tsuji, Ken Uemura, Masahiro Wakita, Tatsuyuki Matsudaira, Tomonori Matsumoto, Shimpei Kawamoto, Akiko Takahashi, Yoshiro Itatani, Hiroaki Miki, Manabu Takamatsu, Kazutaka Obama, Kengo Takeuchi, Makoto Suematsu, Naoko Ohtani, Yosuke Fukunaga, Masashi Ueno, Yoshiharu Sakai, Satoshi Nagayama, Eiji Hara

**Affiliations:** 1 Research Institute for Microbial Diseases (RIMD), Osaka University, Suita, Japan; 2 The Cancer Institute, Japanese Foundation for Cancer Research (JFCR), Tokyo, Japan; 3 Graduate School of Medicine, Kyoto University, Kyoto, Japan; 4 Keio University School of Medicine, Tokyo, Japan; 5 LSI Medience Corporation, Tokyo, Japan; 6 School of Dentistry, Aichi Gakuin University, Nagoya, Japan; 7 Immunology Frontier Research Centre (IFReC), Osaka University, Suita, Japan; 8 The Cancer Institute Hospital, JFCR, Tokyo, Japan; 9 Osaka City University Graduate School of Medicine, Osaka, Japan

**Keywords:** Senescence, Bacteriology, Gastrointestinal cancer

## Abstract

Emerging evidence is revealing that alterations in gut microbiota are associated with colorectal cancer (CRC). However, very little is currently known about whether and how gut microbiota alterations are causally associated with CRC development. Here we show that 12 faecal bacterial taxa are enriched in CRC patients in two independent cohort studies. Among them, 2 *Porphyromonas* species are capable of inducing cellular senescence, an oncogenic stress response, through the secretion of the bacterial metabolite, butyrate. Notably, the invasion of these bacteria is observed in the CRC tissues, coinciding with the elevation of butyrate levels and signs of senescence-associated inflammatory phenotypes. Moreover, although the administration of these bacteria into *Apc*^*Δ14/+*^ mice accelerate the onset of colorectal tumours, this is not the case when bacterial butyrate-synthesis genes are disrupted. These results suggest a causal relationship between *Porphyromonas* species overgrowth and colorectal tumourigenesis which may be due to butyrate-induced senescence.

## Abstract (web summary)

Several bacteria in the gut microbiota have been associated with colorectal cancer (CRC) but it is not completely clear whether they have a role in tumourigenesis. Here, the authors show enrichment of 12 bacterial taxa in two cohorts of CRC patients and that two Porphyromonas species accelerate CRC onset through butyrate secretion.

## Introduction

In concordance with dietary changes and increased longevity over the past decades, the morbidity and mortality rates of colorectal cancer (CRC) have been increasing dramatically and CRC has now become the second most common cancer worldwide^1^. Thus, there is an urgent need for effective measures against CRC, and early detection and prevention of CRC are anticipated. The colon contains the densest metabolically active microbial community in healthy adults, and it is becoming clear that changes in the gut microbiota composition are associated with various diseases, including cancer^2–5^. For example, the introduction of *Fusobacterium nucleatum* or *Bacteroides fragilis* into *Apc*^*Min/+*^ mice accelerated the onset of intestinal tumours^6,7^. Furthermore, it has been reported that *pks*+ *E. coli, Peptostreptococcus anaerobius, Clostridium spp*. and *Bacteroides fragilis* promote colonic tumourigenesis through colibactin production^8,9^, activation of the PI3K-AKT pathways^10^, secondary bile acid production^11^, and *Bacteroides fragilis* toxin production^12^, respectively. Therefore, the use of gut microbiota for CRC screening and/or CRC prevention is attracting keen attention^13,14^. However, since residential areas, medical treatments, and technical protocols strongly affect human gut microbiota variations^15–17^, it is difficult to integrate and compare different sets of published data. More importantly, although many studies have reported that the abundance of certain gut bacteria is increased or decreased in patients with CRC^18–21^, it is not clear which of these gut bacteria are actually involved in the development of CRC except for the aforementioned bacterial species^5,22^. Thus, in order to better understand the mechanism of colorectal carcinogenesis and to develop more effective CRC screening methods, it is necessary to clarify which of the gut bacteria that are increased or decreased in colorectal cancer patients are actually involved in the development of CRC, and how they exert these effects. Toward this end, we need some reliable indicators that can detect potentially oncogenic stimuli.

In response to various oncogenic stimuli, such as the activation of certain oncogenes, the elevation of reactive oxygen species (ROS) levels, or exposure to a myriad of DNA damaging agents, normal cells undergo either apoptotic cell death or irreversible cell-cycle arrest, termed “cellular senescence”^23–28^. Unlike apoptotic cells, however, senescent cells are viable for a long period of time and develop a secretory profile composed of pro-inflammatory and tumour-promoting factors, a typical signature termed the senescence-associated secretory phenotype (SASP)^29–31^. Thus, although cellular senescence initially acts as a tumour suppression mechanism, the accumulation of senescent cells in vivo eventually promotes tumourigenesis through SASP, depending on the biological context^2,32–35^.

Using the cellular senescence response as an indicator to identify gut bacteria with the ability to provoke potentially oncogenic stimuli, we show here that *Porphyromonas gingivalis* and *Porphyromonas asaccharolytica*, which correspond to two of the bacterial taxa enriched in CRC patients and are rarely detectable in healthy individuals (HI), are capable of inducing cellular senescence in cultured cells and intestinal epithelial organoids via secreting butyrate, a short-chain fatty acid (SCFA). Furthermore, *Porphyromonas gingivalis* and, to a lesser extent, *Porphyromonas asaccharolytica* also accelerate colorectal tumour development via butyrate secretion in mice. Our data reveal that the aberrant increase of these butyrate-producing bacteria in the colon is likely to be causally involved in CRC development, depending on the biological context.

## Results

### Identification of gut bacteria enriched in CRC patients

To examine whether and how alterations of the gut bacterial profile in CRC patients are causally involved in CRC development, we first collected stool samples from healthy individuals and patients with CRC, who underwent a colonoscopy to either diagnose CRC or confirm the absence of colonic lesions (Cohort-1). To avoid any unforeseen bias, the stool samples were collected before any treatments were performed, including colonoscopy, antibiotic medication, chemotherapy and radiotherapy (Supplementary Fig. 1a). Furthermore, individuals with digestive tract reconstruction or inflammatory bowel disease, which could influence the gut bacterial composition, were also excluded from this cohort study (Supplementary Fig. 1a). In total, the stool samples from 384 HI and 380 CRC patients, including 63 patients with early CRC (clinical characteristics of participants are shown in Supplementary Fig. 1b and c), were subjected to the meta-sequencing analysis of the variable regions 1 and 2 (V1 and V2) of the bacterial 16S rRNA gene^36^. Amplicon sequence variants (ASVs) were identified from the demultiplexed raw 16S rRNA gene sequences by denoising via the DADA2 pipeline in QIIME2 (version 2019.10)^37^, and were clustered into operational taxonomic units (OTUs) by using the de novo clustering algorithm of VSEARCH at a 97% similarity cutoff. The linear discriminant analysis effect size (LEfSe)^38^ revealed that the abundances of 87 and 24 OTUs were increased and decreased in CRC patients, respectively.

**Fig. 1:**
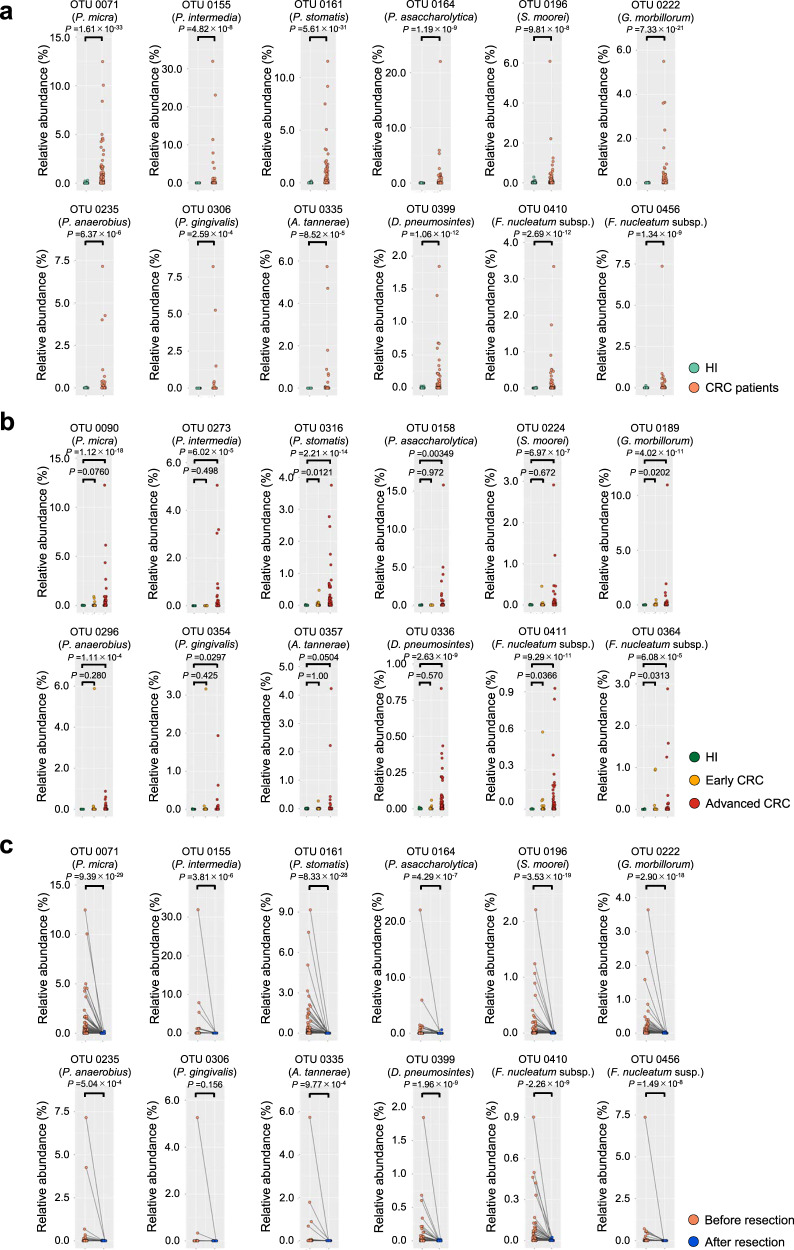
Gut bacteria enriched in CRC patients. **a** Scatter plots showing the abundance (%) of indicated bacterial OTUs enriched in colorectal cancer (CRC) patients of Cohort-1 as determined by 16S rRNA gene-sequencing analysis. The green and red dots represent healthy individuals (HI) or CRC patients, respectively. The name of bacterial species most likely to correspond to each OTU is shown. Statistical significance was determined with a two-tailed Wilcoxon rank-sum test. **b** Scatter plots showing the abundance (%) of indicated bacterial OTUs enriched in CRC patients of Cohort-2 as determined by 16S rRNA gene-sequencing analysis. Green, yellow and red dots represent healthy individuals (HI) or early CRC patients, or advanced CRC patients, respectively. Statistical significance was determined with a Kruskal–Wallis rank-sum test followed by two-tailed pairwise Wilcoxon rank-sum tests. **c** Scatter plots showing the abundances (%) of the indicated OTUs in total gut bacteria of CRC patients of Cohort-1 before (red dots) and after (blue dots) surgical resection of primary tumours. Statistical significance was determined with a two-tailed Wilcoxon signed-rank test. *P* values < 0.05 were considered significant. Source data are provided as a Source Data file.

In order to search for gut bacteria causally associated with CRC development, we next focused on groups of bacterial OTUs that are intensely increased or decreased in CRC patients. We noted that none of the bacterial OTUs that were abundant in HI were commonly absent in CRC patients, but 12 bacterial OTUs that were abundant in CRC patients were rarely present in HI (Fig. 1a). These 12 bacterial OTUs are most likely to correspond to bacterial species associated with periodontal disease^39^, judging from a combination of SILVA database and BLAST database analyses using the V1-V2 region of the bacterial 16S rRNA gene sequence (Supplementary Fig. 1d). To confirm the reproducibility of these results and examine whether these bacterial species were also enriched in early CRC patients, an independent validation cohort (Cohort-2), consisting of 129 HI, 136 early CRC patients and 153 advanced CRC patients, was subjected to a similar analysis (Supplementary Fig. 2). Interestingly, comparable results were obtained using this independent validation cohort, and four of the 12 bacterial OTUs are also increased in a certain number of early CRC patients, although with lower frequencies and numbers than in advanced CRC patients (Fig. 1b). Intriguingly, these bacterial OTUs are no longer detected after surgical resection of CRC (Fig. 1c), further illustrating their strong association with CRC. However, these correlational studies alone cannot distinguish whether the increase of these bacteria, except for *Fusobacterium nucleatum* subsp.^40–42^, in CRC patients is a cause or a consequence of CRC development. Bio-functional analyses are therefore needed to clarify this point.

### *Porphyromonas* species provoke cellular senescence

**Fig. 2:**
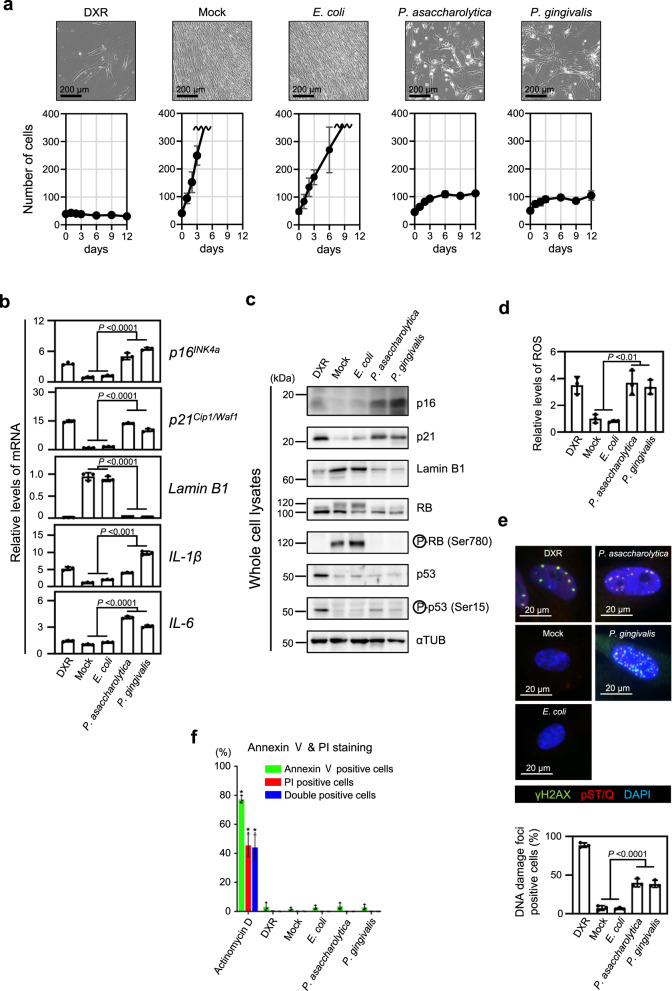
Induction of cellular senescence in fibroblasts by gut bacteria. **a**–**e**, Early passage TIG-3 cells were cultured with tissue culture media containing the indicated bacterial conditioned media or the plain bacterial culture media with (DXR) or without (Mock) doxorubicin at a ratio of 1/30 for 9 days, and then subsequently cultured with plain tissue culture medium for another 3 days. Cell numbers were counted throughout the experiments, and representative photographs of the cells in the indicated culture conditions on day 12 are shown at the top of the panels. These assays were performed in triplicate (both biological and technical replicates) and representative data were shown (**a**). Cells on day 9 were subjected to RT-qPCR analysis for indicated genes (**b**), western blotting analysis using antibodies shown right (**c**), analysis of intracellular ROS levels (**d**) or to immunofluorescence staining for markers of DNA damage (γ-H2AX (green) and pST/Q (red)) and DNA staining with 4′, 6-diamidino-2-phenylindole (DAPI) (blue) (**e**). The histograms indicate the percentage of nuclei that contain more than 3 foci positive for both γ-H2AX and pST/Q staining (**e**). The assay was performed three times and representative data is shown (**c**). **f** TIG-3 cells were cultured with or without (Mock) the indicated bacterial culture supernatants for 9 days, and then subjected to annexin V and Propidium iodide (PI) staining analyses. Actinomycin D treated cells were used as a positive control for apoptotic cells, and doxorubicin (DXR) treated cells were used as a positive control for senescent cells. The histogram shows the percentage of cells that were positive for Annexin V (green bar), PI (red bar) or both (blue bar), respectively. Double positive cells represent apoptotic cells. For all bar graphs, error bars indicate mean ± standard deviation (s.d.) with three biologically independent replicates. Statistical significance was determined with one-way ANOVA followed by Tukey’s test (**b**), (**d**), (**e**) or two-tailed Dunnett’s test for comparing with mock (**f**). *P* values < 0.05 were considered significant. Source data are provided as a Source Data file.

**Fig. 3:**
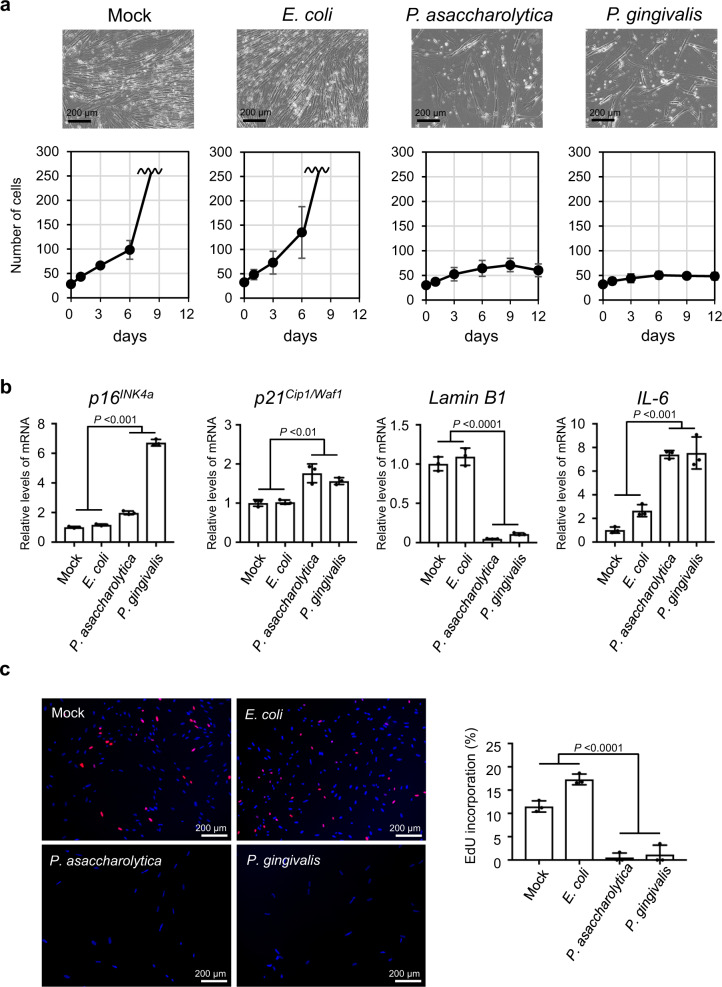
Induction of cellular senescence in intestinal epithelial cells by gut bacteria. **a**, **b** Early passage normal human colonic epithelial cells (CCD 841 CoN) were cultured with tissue culture media containing the indicated bacterial conditioned media or the plain bacterial culture media (Mock) at a ratio of 1/30 for 9 days, changing the medium every 3 days, and then subsequently cultured with plain tissue culture medium for another 3 days. Cell numbers were counted throughout the experiments, and representative photographs of the cells in the indicated culture conditions on day 12 are shown at the top of the panels. These assays were performed in triplicate (both biological and technical replicates) and representative data were shown (**a**). Cells on day 9 were subjected to RT-qPCR analysis for indicated genes (**b**). **c** Early passage normal human colonic epithelial cells (CCD 841 CoN) were cultured with tissue culture media containing the indicated bacterial conditioned media or the plain bacterial culture media (Mock) at a ratio of 1/30 for 9 days, changing the medium every 3 days, and then subsequently cultured with plain tissue culture medium for another 7 days. These cells were then subjected to EdU incorporation analysis. EdU (red) and DNA staining with 4′, 6-diamidino-2-phenylindole (DAPI) (blue) were shown. Representative photographs of the cells in the indicated culture conditions are shown. The histograms indicate the percentages of cells that were positive for EdU. These assays were performed in triplicate (both biological and technical replicates) and representative data were shown (**b**, **c**). For all graphs, error bars indicate mean ± s.d. with three biologically independent replicates. Statistical significance was determined with one-way ANOVA followed by Tukey’s test. *P* values < 0.05 were considered significant (**b**, **c**). Source data are provided as a Source Data file.

Accumulating evidence indicates that cellular senescence is induced by a variety of potentially oncogenic stimuli and suppresses or promotes tumourigenesis, depending on the biological context^25,26,32,33^. Therefore, we decided to use the cellular senescence response as an indicator to determine whether these CRC-enriched bacteria are capable of provoking potentially oncogenic stimuli. Early passage normal human diploid fibroblasts (HDFs), the most commonly used cells in senescence studies, were cultured for 9 days in media containing culture supernatants of the 10 faecal bacterial species corresponding to the bacterial OTUs enriched in CRC patients, except *Fusobacterium nucleatum* subsp., followed by 3 days of culture in medium containing no bacterial culture supernatant (Fig. 2a and Supplementary Fig. 3). Interestingly, similar to doxorubicin (DXR), a DNA damaging agent known to induce cellular senescence^43^, the bacterial culture supernatants of *Porphyromonas asaccharolytica* and *Porphyromonas gingivalis* provoked irreversible cell-cycle arrest in cultured HDFs (Fig. 2a). This was accompanied by typical signatures of cellular senescence, such as the increased expression of senescence-inducing genes (*p16*^*INK4a*^ and *p21*^*Cip1/Waf1*^) and SASP factor genes (*IL-1* and *IL-6*), the reductions of *Lamin B1* expression and the phosphorylated form of pRB, an increase in the phosphorylated form of p53, and the elevation of intracellular ROS levels and DNA damage, without the elevation of an apoptosis marker (Fig. 2b–f). Notably, similar results were also observed in normal human colonic epithelial cells (Fig. 3) and in intestinal epithelial organoids derived from induced pluripotent stem (iPS) cells^44^, except for *p16*^*INK4a*^ expression in intestinal epithelial organoids (Supplementary Fig. 4). Together, these results suggest that these two bacterial species have the ability to induce potentially oncogenic stimuli not only in fibroblasts but also in normal intestinal epithelial cells.

To explore this idea, we next sought to identify the potentially oncogenic stimulatory factor secreted from these two senescence-inducing bacteria. Lipopolysaccharide (LPS), a major component of the outer membrane of Gram-negative bacteria, to which both species belong, reportedly induces osteocyte senescence^45^. However, the senescence-like cell-cycle arrest was induced similarly in primary mouse embryonic fibroblasts regardless of the presence or absence of MyD88 and TRIF, essential downstream mediators of LPS-TLR4 signalling^46^ (Supplementary Fig. 5). Moreover, we could not find the bile acid-inducible (*bai*) genes that are required for bile acid transformation from primary to secondary bile acids^47^ in these two senescence-inducing bacterial genomes (NC_015501.1, NC_010729.1). Thus, although LPS and secondary bile acids are reportedly associated with cellular senescence^2,45^ and CRC^11,48^ in certain settings, it is unlikely that they are potentially oncogenic stimulatory factors secreted from these two *Porphyromonas* species.

### *Porphyromonas* species provoke cellular senescence via butyrate secretion

**Fig. 4:**
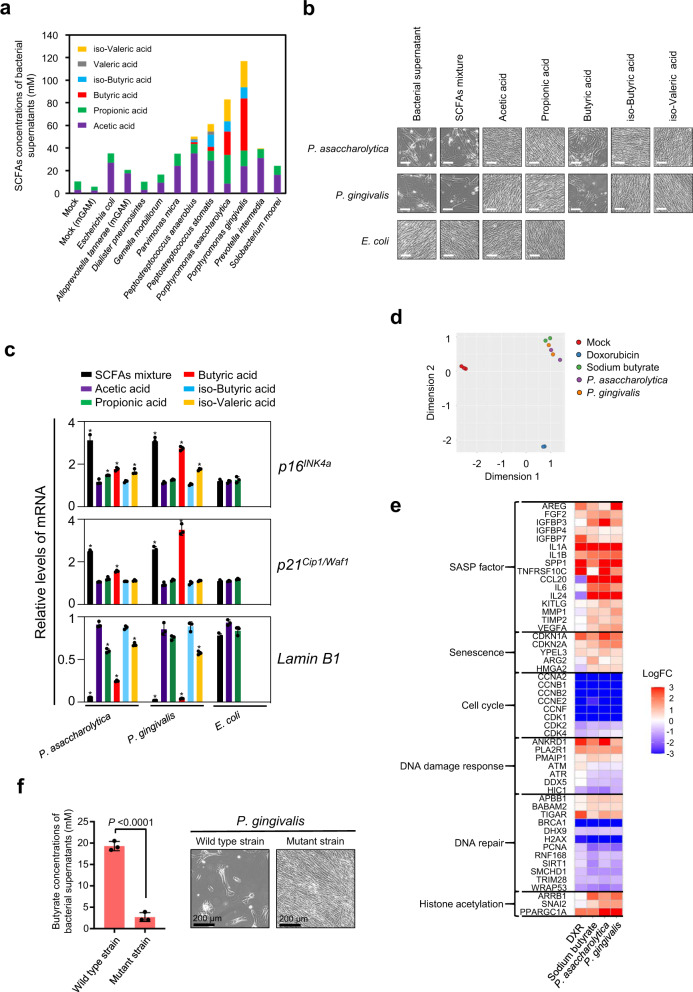
Butyrate provokes cellular senescence in HDFs. **a** The concentrations of SCFAs present in indicated bacterial conditioned media or plain bacterial culture media of GAM (Mock) or modified GAM (Mock-mGAM) were shown. **b**–**e** Early passage TIG-3 cells were cultured for 9 days with tissue culture media containing indicated bacterial conditioned media at the ratio of 1/30 or indicated each SCFA or the mixture of these SCFAs in the same concentrations present in the bacterial conditioned media for 9 days. Representative photographs of the cells in the indicated culture conditions are shown. Scale bars represent 100 μm (**b**). Cells were then subjected to RT-qPCR analysis for indicated genes (one-way ANOVA followed by two-tailed Dunnett’s test for comparing with SCFAs mixture of *E. coli. P* values < 0.05 were considered significant) (**c**), or subjected to RNA sequencing analysis (**d**, **e**). Multi-dimensional scaling (MDS) plots based on the differential gene expression analysis were shown (**d**). Heatmap represents the ratio of gene expression between indicated cells and Mock-treated cells. The colours in the heatmap represent log fold-change (LogFC) relative to mock, with blue indicating three-fold lower expression and red three-fold higher expression (**e**). Three biological replicates of Mock and two biological replicates of the other conditions were analysed (**d**, **e**). The concentrations of doxorubicin (DXR) and Sodium butyrate used were 200 ng/ml or 3 mM, respectively. **f** Measurements of butyrate concentrations in the indicated bacterial conditioned media (left). Wild-type strain represents *P. gingivalis* (ATCC 33277) and Mutant strain represents its butyrate synthesis defective mutant (PGAGU 118)^53^. Error bars indicate mean ± s.d. with three biologically independent replicates. Statistical significance was determined with a two-tailed Student’s *t*-test. *P* value < 0.05 was considered significant. Early passage TIG-3 cells were cultured with tissue culture media containing indicated bacterial conditioned media at the ratio of 1/30 for 9 days and representative photographs of the cells cultured with indicated bacterial conditioned media are shown (right). Source data are provided as a Source Data file.

In seeking alternative factors, we next focused on short-chain fatty acids (SCFAs), major gut bacterial metabolites reported to have various effects on host homoeostasis, including tumourigenesis^22,49^. Among the series of SCFAs tested, the butyrate levels were significantly higher in the bacterial culture supernatants of both *Porphyromonas asaccharolytica* and *Porphyromonas gingivalis*, as compared to those of other CRC-enriched bacteria that failed to induce senescence-like cell-cycle arrest (Fig. 4a). Butyrate reportedly possesses both tumour promotive and suppressive properties through energetic or epigenetic functions, depending on the cell type, duration and amount of exposure^22,49^. Importantly, the treatment of HDFs with the same butyrate concentrations present in the bacterial culture supernatants of *Porphyromonas gingivalis* (1.53 mM) indeed provoked senescence-like phenotypes, but this was not the case with other SCFAs (Fig. 4b, c, Supplementary Fig. 6). Although the same concentration of butyrate present in the culture supernatant of *Porphyromonas asaccharolytica* (0.69 mM) alone weakly induced senescence-like cell-cycle arrest, a co-treatment with the other SCFAs present in this culture supernatant more robustly induced this phenomenon (Fig. 4b, c, Supplementary Fig. 6), implying that other SCFAs may also cooperate with butyrate in provoking senescence-like phenotypes, especially with low butyrate levels. Note that the treatment of normal human colonic epithelial cells with a mixture of SCFAs, at the same concentrations as in the culture supernatants of *Porphyromonas asaccharolytica* and *Porphyromonas gingivalis*, also caused senescence-like cell-cycle arrest (Supplementary Fig. 7). This is somewhat consistent with a previous observation that the CRC-associated microbiome showed an association with the pathways responsible for the conversion of different amino acids to acetate, butyrate, and propionate^19^. Furthermore, a total RNA sequencing analysis revealed that the alterations in the gene expression profiles caused by these bacterial culture supernatants, particularly the SASP factor genes and DNA damage response genes, are more similar to those induced by butyrate as compared to those induced by DXR in cultured HDFs (Fig. 4d, e), implying that butyrate is likely to be a key oncogenic stimulatory factor secreted from these two senescence-inducing bacteria.

Because butyrate induces the expression of the *p16*^*INK4a*^ and *p21*^*Cip1/Waf1*^ genes (Fig. 4c), we next determined whether butyrate promotes cellular senescence through the induction of *p16*^*INK4a*^ and *p21*^*Cip1/Waf1*^ expression. Notably, although the butyrate-treatment induced senescence-like cell-cycle arrest in primary skin fibroblasts prepared from wild-type mice, this was not the case in skin fibroblasts prepared from mice lacking both the *p16*^*INK4a*^ and *p21*^*Cip1/Waf1*^ genes^50^ (Supplementary Fig. 8). These results, together with previous observations that butyrate elicits the epigenetic induction of *p16*^*INK4a*^*, p21*^*Cip1/Waf1*^ and SASP in cultured human cells^51,52^, again implied that butyrate is a major senescence-inducing factor secreted from these bacteria. To further verify this idea, we next tested if the blockade of butyrate production affects the senescence-inducing activity of butyrate-producing bacteria. Indeed, the reduction of butyrate production by deleting PGN_0725, PGN_1341 and PGN_1888, three CoA transferases involved in the last step of butyrate production^53^, rendered the culture supernatant of *Porphyromonas gingivalis* incapable of inducing senescence-responses in HDFs (Fig. 4f, Supplementary Fig. 9). Taken together, these results strongly suggest that butyrate is one of the major potentially oncogenic stimulatory factors secreted from these senescence-inducing bacteria, although we cannot preclude the possibility that other factors may also be involved, depending on the biological context.

### Bacterial invasion and signs of cellular senescence in CRC tissues

**Fig. 5:**
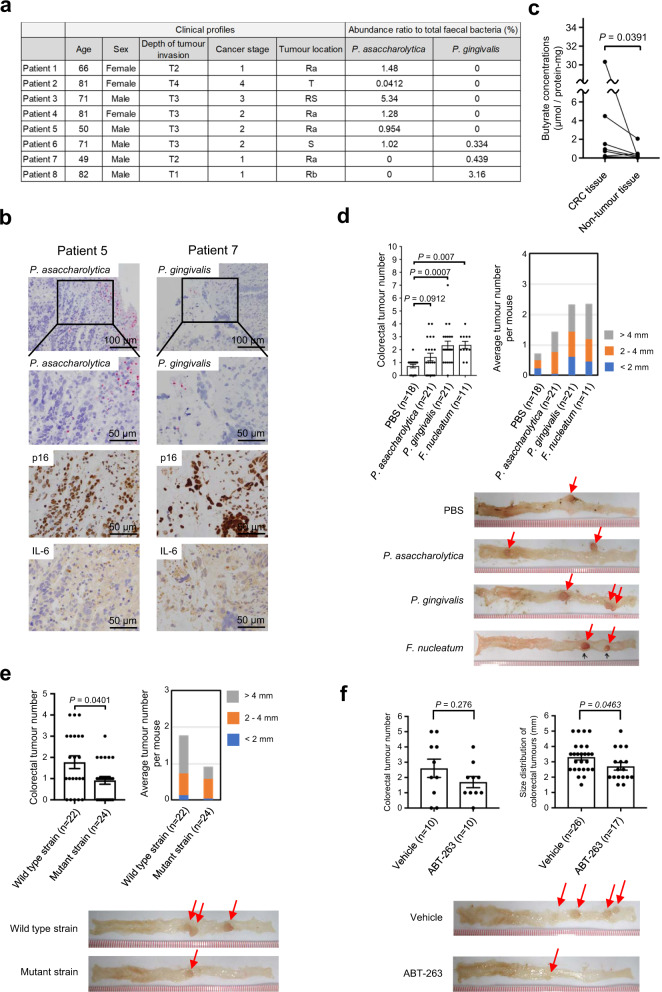
Bacterial invasion and tumour promotion. **a** Clinical information on surgically resected CRC tissues and relative abundances of OTUs corresponding to the two bacterial species (*P. asaccharolytica* and *P. gingivalis*) in the faeces of the patients analysed. **b** Paraffin-embedded CRC tissues were subjected to the in situ hybridization using probes specific to *P. asaccharolytica*, or *P. gingivalis*, or to the immuno-staining analysis using indicated antibodies. These assays were performed in three biologically independent replicates to show representative data. **c** Butyrate concentrations in CRC tissues and paired non-tumour tissues of eight patients. Statistical significance was determined with a two-tailed Wilcoxon signed-rank test. *P* value < 0.05 was considered significant. **d** Colorectal tumour numbers in *Apc*^*Δ14/+*^ mice gavaged with PBS (*n* = 18), *P. asaccharolytica* (*n* = 21), *P. gingivalis* (*n* = 21) or *F. nucleatum* subspecies *nucleatum* (referred to as *F. nucleatum*) (*n* = 11) (left) and the average colorectal tumour numbers with the size distribution per mouse (right). Error bars indicate mean ± standard error of measurement (s.e.m.). Representative macroscopic photographs of the colorectal tumours are shown (bottom). Statistical significance was determined with a Kruskal–Wallis rank-sum test followed by two-tailed pairwise Wilcoxon rank-sum tests. *P* values < 0.05 were considered significant. *F. nucleatum* was used here as a positive control and PBS was used here as a negative control. **e** Colorectal tumour numbers in *Apc*^*Δ14/+*^ mice gavaged with *P. gingivalis* wild-type strain (ATCC 33277) (*n* = 22) or its butyrate synthesis defective mutant strain (PGAGU 118)^53^ (*n* = 24) (upper left) and the average colorectal tumour numbers with the size distribution per mouse (upper right) are shown. Representative macroscopic photographs of the colorectal tumours are shown (bottom). Statistical significance was determined with a two-tailed Wilcoxon rank-sum test. Error bars indicate mean ± s.e.m. *P* value < 0.05 was considered significant. **f** Colorectal tumour numbers in *Apc*^*Δ14/+*^ mice gavaged with *P. gingivalis* wild-type strain with (*n* = 10) or without (*n* = 10) ABT-263 (upper left) and the size distribution of colorectal tumours of indicated mouse are shown (upper right). Representative macroscopic photographs of the colorectal tumours are shown (bottom). Statistical significance was determined with a two-tailed Wilcoxon rank-sum test. Error bars indicate mean ± s.e.m. *P* value < 0.05 was considered significant. *S*ource data are provided as a Source Data file.

To further substantiate this idea, we next sought evidence that cellular senescence occurs in the CRC tissues enriched with these butyrate-producing bacteria. Although high levels (15–25 mM)^54^ of butyrate are present in the colonic lumen, they are reportedly much lower in the intestinal epithelium due to the coverage with a dense layer of mucus, which prevents the translocation of gut bacteria into the underlying tissues^49^. Interestingly, however, an in situ hybridization analysis using species-specific probes revealed that these butyrate-producing bacteria infiltrated the CRC tissues of the patients with faeces enriched with these bacteria (Fig. 5a, b, Supplementary Fig. 10a). Furthermore, to distinguish between *Porphyromonas asaccharolytica* and *Porphyromonas uenonis*, whose 16S rRNA sequences are more than 98% identical, the species-specific gene, *rpoB*, was isolated and sequenced from a patient (Supplementary Figs. 1d and 10b). Notably, the levels of butyrate in tumour regions are substantially higher than those in non-tumour regions, in the same resected specimens (Fig. 5c). These results, in conjunction with the observations that signs of cellular senescence, such as the induction of both p16^INK4a^ and SASP factor (IL-6) expression, were observed in the vicinity of the bacterial invasion within the tumour regions (Fig. 5b), strongly suggest that these two butyrate-producing bacteria are involved in the implementation of cellular senescence in human colorectal tissues. Note that a significant proportion of p16^INK4a^ expressing cells also expresses αSMA (Supplementary Fig. 10c), implying that a senescence-like phenotype may be induced in at least some of the fibroblasts in the tumour microenvironment.

### Butyrate-producing bacteria accelerate the onset of colorectal tumours

Finally, we determined whether these two butyrate-producing bacteria can promote colorectal tumourigenesis in vivo. To this end, we used *Apc*^*Δ14/+*^ mice, in which tumour development is enhanced in the distal colon and rectum^55^, and *Fusobacterium nucleatum* subsp. as a positive control^6^. Notably, the numbers of colorectal tumours developed in *Apc*^*Δ14/+*^ mice administered *Porphyromonas gingivalis* and, to a lesser extent, *Porphyromonas asaccharolytica* by gavage was increased, as compared to that in control (PBS) mice (Fig. 5d). This was accompanied by signs of cellular senescence and increased levels of butyrate in the colorectal tumours developed in *Apc*^*Δ14/+*^ mice administered these bacteria (Supplementary Fig. 11a–d). These results are consistent with the observation that the levels of butyrate in the culture supernatant of *Porphyromonas gingivalis* were higher than those of *Porphyromonas asaccharolytica* (Fig. 4a–c), implying that high levels of bacterial butyrate may promote colorectal tumour development in vivo. To bolster this idea, we next examined whether the reduction of butyrate production by *Porphyromonas gingivalis* decreases the ability to enhance colorectal tumour formation in *Apc*^*Δ14/+*^ mice. Indeed, the number of colorectal tumours developed in *Apc*^*Δ14/+*^ mice administered a butyrate synthesis defective mutant of *Porphyromonas gingivalis*^53^ by gavage was significantly lower than that in *Apc*^*Δ14/+*^ mice administered wild-type *Porphyromonas gingivalis* (Fig. 5e). It is also worth mentioning that the number of colonic tumours produced in mice gavaged with *Porphyromonas gingivalis* tended to be reduced by a treatment with ABT-263, a well-established senolytic drug that preferentially eliminates senescent cells^56^, although this reduction was not statistically significant (Fig. 5f). Notably, the size of the tumours in the group receiving ABT-263 was statistically significantly smaller than that in the group not receiving ABT-263 (Fig. 5f). Moreover, the proportion of p16^INK4a^ expressing fibroblasts (αSMA positive cells) in the tumour regions of mice treated with ABT-263 was lower than that in mice not treated with ABT-263 (Supplementary Fig. 11e). These results, together with previous observations that cellular senescence occurs in human colorectal tumours^30,57^, strongly suggest that the aberrant increases of these butyrate-producing bacteria in the colon are likely to be causally involved in CRC development, possibly by provoking cellular senescence, at least in certain settings.

## Discussion

One of the important questions remaining in the field of gut microbiota in CRC is the issue of whether and how gut microbiota alterations are causally associated with CRC development^5,22^. In this study, we found that 12 faecal bacterial taxa were enriched in CRC patients and rarely detectable in HI, in two different cohort studies (Fig. 1a, b). Although some of the bacterial species corresponding to these bacterial taxa were reportedly increased in CRC patients in several different studies^18–20^, it remained unclear whether these bacterial species, with the exception of *Fusobacterium nucleatum*, were oncogenic or not. Therefore, we explored this topic, using cellular senescence as an indicator. In this study, we used normal lung HDF as a tool to screen for gut bacteria that induce senescence-like phenotypes, because normal lung HDF is the most commonly studied cell in cellular senescence research (Fig. 2). However, it is important to emphasize that we have subsequently confirmed that two bacterial candidates identified using HDFs were able to induce the senescence-like phenotype in intestinal cells (Fig. 3 and Supplementary Fig. 5) and mice (Fig. 5d–f). Note that the SA-β-gal activity, a widely used senescence marker, is also induced when normal cells are rendered quiescent by contact inhibition or serum starvation^58–60^, and is not required for the implementation of cellular senescence^61^. Therefore, in this study, we used other markers known to play key roles in establishing the senescent state, such as DNA damage foci, increased *p21*^*Cip1/Waf1*^ and/or *p16*^*INK4a*^ expression, and decreased lamin B1 expression (Figs. 2, 3, 4, 5, Supplementary Figs. 4, 8, 9 and 11). In several experiments with cultured cells, we also confirmed the irreversibility of cell-cycle arrest, an essential phenotype of cellular senescence (Figs. 2a, 3a, c, Supplementary Figs. 5a and 7). Judging by these markers, we revealed that *Porphyromonas gingivalis* and, to a lesser extent, *Porphyromonas asaccharolytica* have the ability to elicit senescence-like phenotypes and colorectal tumour development via butyrate secretion (Figs. 4 and 5).

Several reports have shown that butyrate also possesses tumour suppression properties, likely through its action as a histone deacetylase (HDAC) inhibitor^22,49^. On the other hand, numerous studies have demonstrated that butyrate promotes colorectal carcinogenesis in different CRC animal models and humans^22,49^. This discrepancy in the effects of butyrate on tumourigenesis has been termed the butyrate paradox, and may be reconciled by the butyrate concentration^62^. For instance, although a low dose of butyrate promotes tumourigenesis, a high dose inhibits it^49^. However, recent studies have shown that relatively high concentrations of butyrate can also enhance the development of CRC^63^ and liver cancer^64^, making it difficult to describe the butyrate paradox simply by differences in butyrate concentrations. Thus, considering the two faces of cellular senescence (tumour suppression and tumour promotion)^32,33^ and our findings, it is tempting to speculate that the butyrate paradox can also be explained by the opposing effects of cellular senescence on tumourigenesis, at least in certain settings.

It is worth mentioning that the 12 faecal bacterial taxa enriched in the faeces of CRC patients and rarely detectable in those of HI apparently correspond to bacterial species associated with periodontal disease^39^ (Supplementary Fig. 1d). These results are consistent with previous observations that many of the bacteria that are enriched in the faeces of CRC patients are associated with typical oral commensals^18–21^. It is further interesting to note that a recent large cohort study revealed that women with a small number of teeth and moderate or severe periodontal inflammation had up to a 48% increased risk of developing CRC^65^. Collectively, although we still know very little about the mechanism of ectopic colonisation of the gut by oral bacteria, it is clear that a bacterial link exists between periodontal disease and CRC.

Senescent cells can recruit various immune cells, such as macrophages, natural killer (NK) cells, neutrophils and T-lymphocytes via SASP, and the excessive accumulation of immune cells is known to cause chronic inflammation that may promote carcinogenesis^66^. Furthermore, cellular senescence reportedly impairs the tight junction and barrier integrities of epithelial cells^67^. Thus, it is also tempting to speculate that the aberrant increase of butyrate-producing bacteria in the colon may contribute to CRC development, possibly through promoting the infiltration of gut bacteria and immune cells into the gut epithelial barrier, at least in certain settings. However, we cannot exclude the possibility that some bacteria may promote CRC development without eliciting the senescence response^10,41^. Moreover, the mechanism of induction of cellular senescence in vivo may be somewhat different from that in vitro. Thus, further studies are needed to understand the mechanisms of the gut bacterial link to CRC development. Nevertheless, while additional mechanisms may be at play here, our data raise the possibility that a subset of gut bacteria increased in CRC patients has the potential to give rise to cellular senescence and tumourigenesis through butyrate secretion. These findings expand our understanding of whether and how gut microbiota alterations are causally associated with CRC development and open up possibilities for its control.

## Methods

### Human faecal sample collection

Faeces were collected from colorectal cancer patients (CRC patients) and healthy individuals (HI) who visited the Cancer Institute Hospital of JFCR from December 2013 to March 2015 (Cohort-1) and January to September 2017 (Cohort-2) using a faecal sampling tool (TechnoSuruga Laboratory). Patients with a history of inflammatory bowel disease, those who had undergone gastrointestinal reconstructive surgery, those with severe hepatic dysfunction, those who received antibiotic treatment within 1 month, those who had a faecal sample within 3 days of colonoscopy, or those for whom complete personal information was not available were excluded from CRC patients and HI. Patients who received chemotherapy or radiation therapy before faecal sample collection, who had faecal samples collected after endoscopic tumour resection, or patients with CRC whose tumours were not adenocarcinomas (e.g., squamous cell carcinoma) were excluded. HI with a history of colorectal cancer or with malignancy at the time of stool collection were excluded. Informed consent was obtained from all participants for the use of anonymized samples and the publication of the patients’ clinical information in accordance with the protocol approved by the ethics committee of the JFCR. The tumour profiles of CRC patients were classified based on the Third English Edition of the Japanese Classification of Colorectal, Appendiceal and Anal Carcinoma. The statistical significance of clinical data was determined using a Wilcoxon rank-sum test (Cohort-1), a Kruskal–Wallis rank-sum test (Cohort-2) or a Fisher’s exact test (Cohort-1 and 2).

### 16S rRNA gene-sequencing analysis and microbiome analysis

Bacterial DNA extraction from faecal samples was performed using QIAamp Fast DNA Stool Mini Kit (QIAGEN) (samples of Cohort-1) or a Magtration System 12GC (Precision System Science, Japan) in TechnoSuruga Laboratory (samples of Cohort-2 and samples of Cohort-1 collected after tumour resection). Twenty-five cycles of amplification of the V1-V2 region of the bacterial 16S rRNA gene were performed using KOD FX Neo DNA polymerase (TOYOBO) with universal 16S rRNA primers followed by the secondary amplification adding the Illumina flow cell adapters and indices. The PCR primers used are shown in Supplementary Table 1. Meta-16S rRNA gene sequencings were carried out per 96 samples on the Illumina Miseq platform (Illumina) using Miseq Reagent Kit V2 (Illumina) (paired-end, 225 cycles × 2). Sequencing reads were processed according to the QIIME2 (version 2019.10) pipeline^37^. Fastq files were de-noised with DADA2 plugin and amplicon sequence variants (ASVs) were counted. Subsequently, de novo clustering was performed on ASVs using the VSEARCH plugin to obtain operational taxonomic units (OTUs) with a similarity of more than 97%. These processes were performed separately for samples from Cohort-1 and Cohort-2. Open-reference clustering based on OTUs detected from Cohort-1 samples collected before tumour resection was performed on samples collected after tumour resection. Finally, the OTU counts were converted to relative abundance per sample. Phylogenetic classification of the detected OTUs was performed by a Naive Bayes classifier trained on the SILVA132 database (QIIME-compatible SILVA releases) in the QIIME2 pipeline. Identification of the specific bacterial species corresponding to each OTU was performed by using the 16S rRNA database provided by the National Center for Biotechnology Information (NCBI) (last modified at 2020/05/03) and a similarity search with BLAST+ (version 2.9.0). OTUs with significant differences in the relative abundance of Cohort-1 HI and CRC patients were detected by the linear discriminant analysis effect size (LEfSe) (version 1.0.8.post1)^38^. OTUs detected in only one sample were removed prior to analysis. From the OTUs selected by LEfSe, OTUs that were only increased in CRC patients and rarely detected by HI were picked. The relative abundance of these OTUs was compared between HI, early CRC patients, and advanced CRC patients (Cohort-2). The relative abundances of OTUs in CRC patients of Cohort-1 were also compared before and after tumour resection.

### Bacterial strain and culture conditions

*Alloprevotella tannerae* JCM 16135, *Dialister pneumosintes* JCM 10004, *Fusobacterium nucleatum* subsp. *nucleatum* JCM 8532, *Gemella morbillorum* JCM 12968, *Parvimonas micra* JCM 12970, *Peptostreptococcus anaerobius* JCM 1769, *Peptostreptococcus stomatis* JCM 15636, *Porphyromonas asaccharolytica* JCM 6323, *Porphyromonas gingivalis* JCM 8525, *Prevotella intermedia* JCM 11150 and *Solobacterium moorei* JCM 10645 were provided by the RIKEN BRC through the National Bio-Resource Project of the MEXT, Japan. *Escherichia coli* DH5α (DNA-903) was purchased from TOYOBO. *A. tannerae* was anaerobically cultured with modified Gifu Anaerobic Medium (mGAM). Other 11 bacteria were anaerobically cultured with Gifu Anaerobic Medium (GAM). For the preparation of bacterial conditioned medium, each bacterium was cultured for 2 to 3 days. *P. gingivalis* ATCC 33277 and its butyrate synthesis defective mutant strain PGAGU 118^53^ which lacks three butyryl CoA:acetate CoA transferase, PGN_0725, PGN_1341 and PGN_1888 were anaerobically cultured with GAM to an OD600 value of 3.5. Cultured medium containing each bacterium was collected and centrifuged at 10,000 × *g* for 10 min. After filtration through a 0.22-μm mesh, the conditioned medium of each bacterium was collected and stocked at −80 °C until use. The SCFAs concentrations were measured immediately after the collection of the cell-free bacterial supernatants. For oral gavage to mice, *F. nucleatum* subsp. *nucleatum*, *P. asaccharolytica*, *P. gingivalis* JCM 8525, *P. gingivalis* ATCC 33277 and its butyrate synthesis defective mutant strain PGAGU 118 were anaerobically cultured in GAM and collected in a logarithmic growth phase. The colony-forming unit (CFU) was measured by serial dilution and plating on GAM agar.

### Cell culture

Early passage (< 45 population doublings) human diploid fibroblasts (HDFs) TIG-3 cell, early passage mouse embryonic fibroblasts (MEFs) isolated from wild-type mice or mice lacking both *Myd88* and *Trif* genes (*Myd88*^−/−^ *Trif*^−/−^ mouse) (Oriental BioService), and early passage skin fibroblasts isolated from wild-type mice or mice lacking both *p16*^*INK4a*^ and *p21*^*Cip1/Waf1*^ (*p16*^−/−^*, p21*^*−/−*^ mouse)^50^ were cultured in Dulbecco’s modified Eagle medium (DMEM) supplemented with 10% foetal bovine serum (FBS). Normal human colonic epithelial cells (CCD 841 CoN) were cultured in Eagle’s minimum essential medium (EMEM) supplemented with 10% FBS. In some experiments, cells were cultured in a tissue culture medium containing 1/30 volume of filtered bacterial culture supernatant or SCFA at the indicated concentrations for 9 days, with the medium changed every 3 days. These cells were then cultured in plain DMEM supplemented with 10% FBS for an additional 3 or 7 days.

### iPSC-derived intestinal organoid culture

Intestinal organoids were differentiated from the human-induced pluripotent stem cell (iPSC) line TkDN4-M supplied by the University of Tokyo^44,68^. Conditioned medium containing mouse Wnt3a, human R-spondin 1, human noggin, and human hepatocyte growth factor (WRNH conditioned medium) was prepared by incubating WRNH-expressing L cells for 72 h^44^. Approximately 300 cells were resuspended in 25 μl Matrigel with 20% organoid culture medium (advanced Dulbecco’s modified Eagle medium/F12 (Thermo Fisher Scientific) supplemented with 25% WRNH conditioned medium, 10 mM HEPES (pH 7.3) (Thermo Fisher Scientific), 2 mM GlutaMAX^TM^ (Thermo Fisher Scientific), 1× B-27 (Thermo Fisher Scientific), 50 ng/mL murine epidermal growth factor (mEGF) (Peprotech), 10 μM p38 Mitogen-activated protein kinase (MAPK) inhibitor (SB202190; Sigma-Aldrich), 500 nM transforming growth factor β receptor inhibitor (A83-01; Tocris) and 100 units/mL penicillin plus 100 μg/mL streptomycin) per well in 48-well culture plate at the passage and cultured with organoid culture medium plus 10 μM ROCK inhibitor Y-27632 (Wako)^44^. Two days after passage, organoids were cultured with organoid culture medium containing bacterial conditioned medium of *P. asaccharolytica*, *P. gingivalis* JCM 8525 or *E. coli*, or plain bacterial culture medium (GAM) at the ratio of 1/30. Each medium was changed every 2 or 3 days.

### Quantitative real-time PCR analysis

Total RNA was extracted using TRIzol (Thermo Fisher Scientific) or RNeasy mini kit (Qiagen) according to the manufacturer’s protocol. cDNA was synthesized using a PrimeScript RT reagent kit (Takara Bio Inc.). Quantitative real-time RT-PCR was performed on a StepOnePlus PCR System (Applied Biosystems) using SYBR Premix EX Taq (Takara Bio Inc.). The mRNA expression levels of each gene were calculated relative to β-actin expression levels. The PCR primer sequences used are shown in Supplementary Table 1.

### Immunoblotting

Proteins were extracted using RIPA buffer with 1% Protease inhibitor cocktail (Nacalai Tesque). After determination of the protein concentration using Protein Quantification Assay (Takara Bio Inc.), all samples were denatured in Laemmli sample buffer for 5 min at 100 °C. The denatured samples were separated by SDS-polyacrylamide gel electrophoresis and transferred onto a polyvinylidene difluoride membrane (EMD Millipore). After blocking with 5% milk or 5% BSA, the membranes were incubated with the primary antibodies as follows: p16^INK4a^ (1:1000, IBL, 11104), p21^Cip1/Waf1^ (1:1000, Cell Signaling Technology, 2947), Lamin B1 (1:5000, Abcam, ab16048), RB (1:500, Santa Cruz, sc-102), phospho-RB (Ser780) (1:1000, Cell Signaling Technology, 9307), p53 (1:1000, Santa Cruz, sc-6243), phospho-p53 (Ser15) (1:1000, Cell Signaling Technology, 9284) and α-Tubulin (1:2000, Sigma-Aldrich, T9026). The membranes were then incubated with the secondary antibodies as follows: anti-mouse IgG (1:2000, Cell Signaling Technology, 7076), anti-rabbit IgG (1:2000, Cell Signaling Technology, 7074) and visualized with Amersham ECL prime/select (GE Healthcare), followed by detection with chemiluminescence using LAS-3000mini imaging system (Fujifilm) and by analysis of data using Multi Guage V3.1 (Fujifilm). Uncropped and unprocessed scans of the blots are included in the Source data file.

### ROS measurement

To assess the levels of intracellular ROS generation, cells were incubated with 20 μM DCF-DA (Calbiochem) at 37 °C for 15 min. The peak excitation wavelength for oxidized DCF (488 nm) and that for emission (525 nm) were measured by ARVO MX/Light 1420 Multilabel/Luminescence Counter (PerkinElmer).

### Immunofluorescence analysis

Immunofluorescence analysis of TIG-3 cells and iPSC-derived intestinal organoids was performed using antibodies against γ-H2AX (Millipore, 05-636, 1:1000 dilution), phospho-(Ser/Thr) ATM/ATR substrate (pST/Q) (Cell Signaling Technology, 2851, 1:500 dilution) or Cleaved Caspase-3 (Asp175) (Cell Signaling Technology, 9661, 1:100 dilution). The secondary antibodies, donkey anti-mouse IgG Alexa Flour 488 (Thermo Fisher Scientific, A-21202), donkey anti-rabbit IgG Alexa Fluor 594 (Thermo Fisher Scientific, A-21207) or donkey anti-rabbit IgG Alexa Flour Plus 555 (Thermo Fisher Scientific, A-32794) was used with 1:1000 dilution for primary antibodies against γ-H2AX, pST/Q or Cleaved Caspase-3, respectively. DNA was stained with 4′,6-diamidino-2-phenylindole (DAPI) (Dojindo). Fluorescence images were obtained with a Fluorescence Microscope BZ-X710 (KEYENCE) or FLUOVIEW FV3000 confocal laser scanning microscope (OLYMPUS) and analysed using Image J 1.53e.

### Apoptosis analysis

Apoptosis analysis of TIG-3 was performed using Annexin V-FITC (MBL Life Science, 4700-100) and Propidium iodide (PI) solution (Sigma-Aldrich, P4864) according to the manufacturer’s protocol. DNA was stained with DAPI (Dojindo).

### EdU incorporation assay

Cell proliferation was evaluated using Click-iT™ Plus EdU Cell Proliferation Kit for Imaging Alexa Fluor™ 555 dye (Thermo Fisher Scientific, C10638) according to the manufacturer’s protocol.

### RNA sequencing analysis

RNA sequencing library was prepared using TruSeq stranded mRNA Sample Prep Kit (Illumina) according to the manufacturer’s protocol. Sequencing was carried out on the Illumina HiSeq 2500 platform (Illumina) (a 75-base, single-end). The quality of the sequencing data was confirmed by FastQC (version 0.11.8). Sequencing reads were mapped to the GRCh38 human reference genome (Ensembl release 100) using STAR (version 2.7.4a). Counting of reads per gene was performed by HTSeq (version 0.12.4). Differential gene expression analysis was performed using edgeR (version 3.30.3). The gene symbols were based on org.Hs.eg.db (version 3.11.4). The statistical significance was determined with quasi-likelihood *F*-tests and the *P* values were adjusted by the Benjamini–Hochberg false-discovery rate (FDR) correction at 0.05.

### Animal experiment

C57BL/6J *Apc*^*Δ14/+*^ mice^55^ which express a mutant adenomatous polyposis coli gene with deletion of exon 14 at the age of 6 weeks were subjected to oral gavage with 200 μl of PBS or PBS containing bacteria (*F. nucleatum* subsp. *nucleatum*, *P. asaccharolytica*, *P. gingivalis* JCM 8525, *P. gingivalis* ATCC 33277^53^, butyrate synthesis defective mutant *P. gingivalis* PGAGU 118)^53^ at 1 × 10^8^ CFU every other days for 8 weeks with or without ABT-263 (100 mg/kg or Vehicle p.o. five times a week for a total of 4 weeks with 2 non-administration periods for 1 week). At the end of the experiment, the mice were euthanized and the colon tissue was opened longitudinally. The number of tumours in the colon of each mouse was counted. The colonic tissue was then swiss-rolled and fixed with 4% paraformaldehyde, followed by a paraffin burial technique. Mice were maintained under temperature- and humidity-controlled conditions and were exposed to on a 12-h light–dark cycle. All mouse experiments were approved by the Animal Research Committee of Research Institute for Microbial Diseases, Osaka University.

### Histology, immunohistochemistry and immunofluorescence

Samples of human colorectal cancer tissues and murine colon tissues were sectioned in 2-μm thick by a microtome, deparaffinized in xylene, rehydrated, and then stained with haematoxylin and eosin. For immunohistochemistry of murine colon tissues, deparaffinized and rehydrated sections were treated with microwave-oven heating in 10 mM citrate buffer (pH 6.0) for antigen retrieval and blocked using Streptavidin/Biotin Blocking Kit (Vector Laboratories). The primary antibodies used were as follows: p16 (1:200, Abcam, ab211542), p21 (1:200, Abcam, ab107099) and IL-6 (1:400, Abcam, ab6672). The secondary antibodies used were as follows: goat anti-rabbit IgG (1:1000, Vector Laboratories, BA-1000), goat anti-rat IgG (1:1000, Vector Laboratories, BA-9400). For immunohistochemistry of human colorectal cancer tissues, all procedures were carried out on the Leica Bond III Automated IHC and ISH system (Leica Microsystems) with the primary antibodies as follows: p16 (1:3000, Abgent, ALS16384) and IL-6 (1:100, Abcam, ab6672). Optical images were obtained with a BX53 Upright Microscope (OLYMPUS) and analysed using cellSens Standard 2.1 (OLYMPUS). Immunofluorescence of murine colon tissues was performed using the primary antibodies against ɑ-SMA (1:2000, Sigma-Aldrich, A5228) and p16 (1:200, Abcam, ab211542). Immunofluorescence of human colorectal cancer tissues was performed using the primary antibodies against ɑ-SMA (1:100, Abcam, ab125057) and p16 (1:1000, Abcam, ab81278). For immunofluorescence analysis of murine and human tissues, the secondary antibodies used were as follows: donkey anti-mouse IgG Alexa Flour 488 (1:1000, Thermo Fisher Scientific, A-21202), donkey anti-rabbit Alexa Flour Plus 555 (1:1000, Thermo Fisher Scientific, A-32794). The nuclei were stained with DAPI (Dojindo). Fluorescent images were obtained with a Fluorescence Microscope BZ-X710 (KEYENCE) and analysed using Image J 1.53e. The study using human clinical samples was approved by the Ethics Committee of the JFCR and Osaka University and all participants provided written informed consent.

### In situ hybridization

Chromogenic RNA in situ hybridization targeting to specific bacterial species (*P. asaccharolytica* and *P. gingivalis*) in colorectal cancer tissues was performed using RNAscope® 2.5 HD Reagent Kit- RED (Advanced Cell Diagnostics (ACD), 322350) and Fast Red according to the manufacturer’s protocol^42^. The probes were as follows: RNAscope® Probe-B-P.asaccharolytica-23srRNA-O1 (ACD, 838331) targeting *P. asaccharolytica* (286-336 of NR_076875.1) and RNAscope® Probe-B-P.gingivalis-23srRNA (ACD, 843341) targeting *P. gingivalis* (122-162 of NR_076276.1). The study was approved by the Ethics Committee of the JFCR and Osaka University and all participants provided written informed consent.

### Measurement of SCFAs

SCFA concentrations in bacterial culture supernatants were measured by ion-exclusion high-performance liquid chromatography^69^ in the Institute of Nutrition and Pathology Inc. Concentrations of butyrate in CRC tissues and paired non-tumour tissues were measured by liquid chromatography-electrospray ionization-tandem mass spectrometry (LC-ESI-MS/MS) with chemical derivatization^70^.

### DNA cloning and sequencing

Amplification of *rpoB* gene in faecal samples was performed using KOD FX Neo DNA polymerase (TOYOBO) with primers shown in Supplementary Table 1. PCR products were cloned into pGEM-T vector (Promega, A3600). DNA was extracted using FavorPrep^TM^ Plasmid DNA Extraction Mini Kit (Favorgen, FAPDE001-1) and subjected to sequencing using Applied Biosystems 3500xL Genetic Analyzer (Thermo Fisher Scientific). Sequencing data of *rpoB* gene was compared with *rpoB* gene of *P. asaccharolytica* (CP002689.1) and *P. uenonis* (NZ_BAJM01000009) using A plasmid Editor (version 2).

### Statistics

Statistical analysis was performed using R (version4.0.2) (Fig. 1a–c, Supplementary Figs. 1b, 2b) or Graph Pad Prism 9 (Figs. 2b, d–f, 3b, c, 4c, f, 5c–f, Supplementary Figs. 4b-e, 8b, 8c, 9b, 11c-e). Wilcoxon rank-sum test (Figs. 1a, 5e, f, Supplementary Fig. 11e), Wilcoxon signed-rank test (Figs. 1c, 5c, Supplementary Fig. 11d), or Student’s *t*-test (Fig. 4f, Supplementary Figs. 8b, c, 9b, 11c) was performed to compare the variables of the two sample groups. To compare the variables of the multiple sample groups, Kruskal–Wallis rank-sum test followed by pairwise Wilcoxon rank-sum test with the adjustment of *P* values by Benjamini–Hochberg FDR correction at 0.05 (Figs. 1b, 5d) or one-way analysis of variance followed by Tukey’s test (Figs. 2b, d, e, 3b, c, Supplementary Fig. 4b-d) or Dunnett’s test (Figs. 2f, 4c, Supplementary Fig. 4e) was performed. Data are expressed as the mean ± s.d. or mean ± s.e.m. as indicated in the figure legends. Statistical tests were two-tailed and *P* < 0.05 was considered significant.

### Reporting summary

Further information on experimental design is available in the Nature Research Reporting Summary linked to this paper.

## Supplementary information

Supplementary Information

Reporting summary

## Data Availability

Microbiome analysis (bacterial 16S rRNA gene meta-sequence) data and RNA sequencing (TIG-3 cells) data generated in this study have been deposited in the DNA Data Bank of Japan (DDBJ) with the accession codes DRA011735 or DRA011736, respectively (https://www.ddbj.nig.ac.jp). The deposited data are available in NCBI under accession numbers DRA011735 and DRA011736. The databases referred in microbiome analysis are as follows: SILVA (https://www.arb-silva.de/) and National Center for Biotechnology Information (NCBI) (https://www.ncbi.nlm.nih.gov/). Bacterial gene data mentioned in the text are available in NCBI under accession numbers NC_015501.1, NC_00729.1, CP002689.1, NZ_BAJM01000009, PGN_0725, PGN_1341 and PGN_1888. The database referred in RNA sequencing analysis is available in Ensembl (https://www.ensembl.org/). Uncropped and unprocessed scans of the blots are included in the Source data file. Source data are provided with this paper.

## Source data

Source Data
