# Supplementary material for: Gut bacteria identified in colorectal cancer patients promote tumourigenesis via butyrate secretion
Source: Nat Commun. 2021 Sep 28;12:5674. doi: 10.1038/s41467-021-25965-x (PMC8479117; doi:10.1038/s41467-021-25965-x)
Supplement: Supplementary file 1 — Supplementary Information [file 41467_2021_25965_MOESM1_ESM.pdf]

Supplementary Table 1. Primers used in this study.

Primers for 16S rRNA gene-sequencing		
1st PCR		
Primer name		Sequence
27Fmod	Forward	ACACTCTTTCCCTACACGACGCTCTTCCGATCTNNAGRGTTTGATYMTGGCTCAG
338R	Reverse	GTGACTGGAGTTCAGACGTGTGCTCTTCCGATCTNNTGCTGCCTCCCGTAGGAGT
2nd PCR		
Primer name		Sequence
D501	Forward	AATGATACGGCGACCACCGAGATCTACACTATAGCCTACACTCTTTCCCTACACGACGCTCTTCCGATCT
D502	Forward	AATGATACGGCGACCACCGAGATCTACACATAGAGGCACACTCTTTCCCTACACGACGCTCTTCCGATCT
D503	Forward	AATGATACGGCGACCACCGAGATCTACACCCTATCCTACACTCTTTCCCTACACGACGCTCTTCCGATCT
D504	Forward	AATGATACGGCGACCACCGAGATCTACACGGCTCTGAACACTCTTTCCCTACACGACGCTCTTCCGATCT
D505	Forward	AATGATACGGCGACCACCGAGATCTACACAGGCGAAGACACTCTTTCCCTACACGACGCTCTTCCGATCT
D506	Forward	AATGATACGGCGACCACCGAGATCTACACTAATCTTAACACTCTTTCCCTACACGACGCTCTTCCGATCT
D507	Forward	AATGATACGGCGACCACCGAGATCTACACCAGGACGTACACTCTTTCCCTACACGACGCTCTTCCGATCT
D508	Forward	AATGATACGGCGACCACCGAGATCTACACGTACTGACACACTCTTTCCCTACACGACGCTCTTCCGATCT
D701	Reverse	CAAGCAGAAGACGGCATACGAGATCGAGTAATGTGACTGGAGTTCAGACGTGTGCTCTTCCGATCT
D702	Reverse	CAAGCAGAAGACGGCATACGAGATTCTCCGGAGTGACTGGAGTTCAGACGTGTGCTCTTCCGATCT
D703	Reverse	CAAGCAGAAGACGGCATACGAGATAATGAGCGGT

		GACTGGAGTTCAGACGTGTGCTCTTCCGATCT
D704	Reverse	CAAGCAGAAGACGGCATACGAGATGGAATCTCGT GACTGGAGTTCAGACGTGTGCTCTTCCGATCT
D705	Reverse	CAAGCAGAAGACGGCATACGAGATTTCTGAATGT GACTGGAGTTCAGACGTGTGCTCTTCCGATCT
D706	Reverse	CAAGCAGAAGACGGCATACGAGATACGAATTCGT GACTGGAGTTCAGACGTGTGCTCTTCCGATCT
D707	Reverse	CAAGCAGAAGACGGCATACGAGATAGCTTCAGGT GACTGGAGTTCAGACGTGTGCTCTTCCGATCT
D708	Reverse	CAAGCAGAAGACGGCATACGAGATGCGCATTAGT GACTGGAGTTCAGACGTGTGCTCTTCCGATCT
D709	Reverse	CAAGCAGAAGACGGCATACGAGATCATAGCCGGT GACTGGAGTTCAGACGTGTGCTCTTCCGATCT
D710	Reverse	CAAGCAGAAGACGGCATACGAGATTTCGCGGAGT GACTGGAGTTCAGACGTGTGCTCTTCCGATCT
D711	Reverse	CAAGCAGAAGACGGCATACGAGATGCGCGAGAG TGACTGGAGTTCAGACGTGTGCTCTTCCGATCT
D712	Reverse	CAAGCAGAAGACGGCATACGAGATCTATCGCTGT GACTGGAGTTCAGACGTGTGCTCTTCCGATCT
Primers for quantitative real-time PCR		
Gene name		sequence
human *β-actin*	Forward	TGGATCAGCAAGCAGGAGTATG
human *β-actin*	Reverse	GCATTTGCGGTGGACGAT
human $p16^{INK4a}$	Forward	ACCAGAGGCAGTAACCATGC
human $p16^{INK4a}$	Reverse	AAGTTTCCCGAGGTTTCTCA
human $p21^{Cip1/Waf1}$	Forward	AGCGATGGAACTTCGACTTTG
human $p21^{Cip1/Waf1}$	Reverse	CGAAGTCACCCTCCAGTGGT
human *Lamin B1*	Forward	GATTGCCCAGTTGGAAGCCT
human *Lamin B1*	Reverse	TGGTCTCGTTAATCTCCTCTTCATACA
human *IL-1β*	Forward	CTGTCCTGCGTGTTGAAAGA
human *IL-1β*	Reverse	TTGGGTAATTTTTGGGATCTACA
human *IL-6*	Forward	AAAGAGGCACTGGCAGAAAA
human *IL-6*	Reverse	TTTCACCAGGCAAGTCTCCT
mouse *β-actin*	Forward	GATGACCCAGATCATGTTTGA
mouse *β-actin*	Reverse	GGAGAGCATAGCCCTCGTAG

mouse *p16*^{INK4a}	Forward	GAACTCTTTCGGTCGTACCC
mouse *p16*^{INK4a}	Reverse	CGAATCTGCACCGTAGTTGA
Primers for DNA cloning and sequencing		
Gene name		sequence
rpoB	Forward	GAGCCAATCGTAAGGGAGGTAT
rpoB	Reverse	AGTCCGTTGCCGTGGTCTTGTG

Supplementary Fig. 1

a

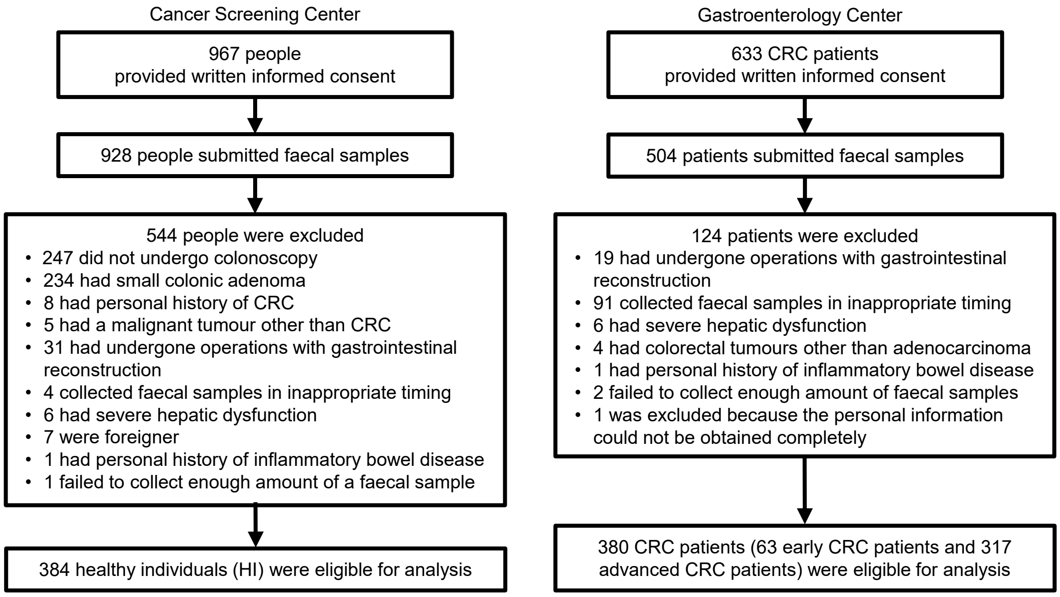

b

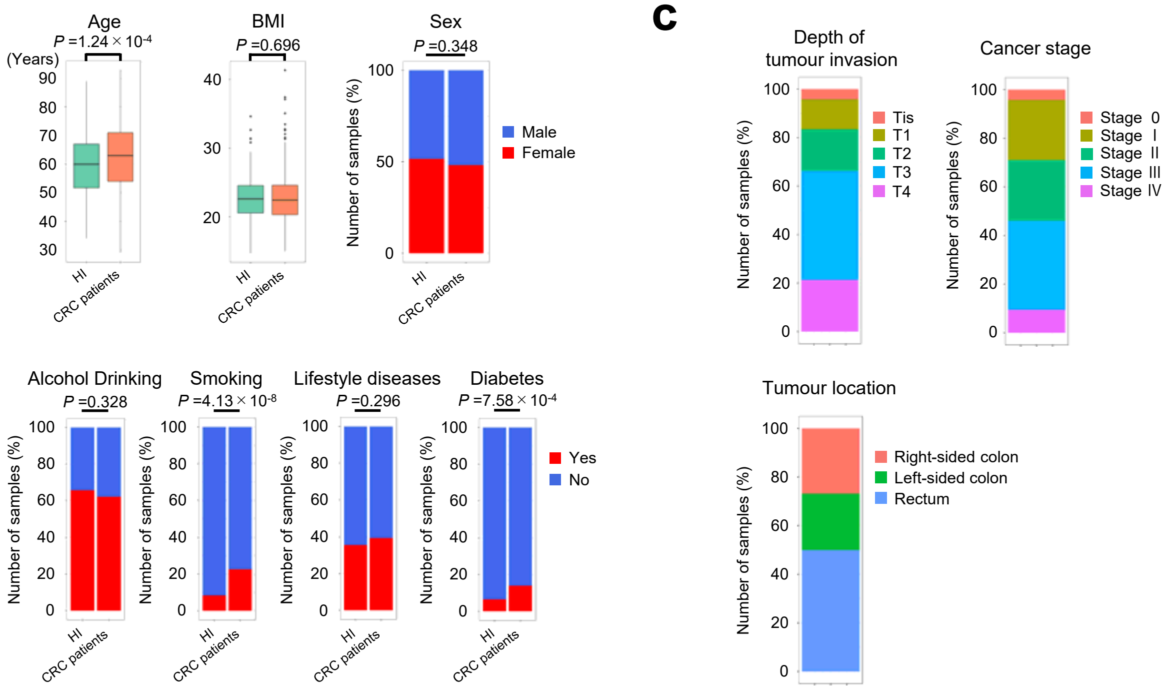

d

OTU ID	Results of the sequence similarity search using BLAST			LDA socre
	Bacterial species most likely to match	Accession no.	Identity	
OTU 0071	*Parvimonas micra* JCM 12970	NR_114338.1	100.0%	3.159
OTU 0155	*Prevotella intermedia* JCM 12248	NR_113106.1	100.0%	3.108
OTU 0161	*Peptostreptococcus stomatis* W2278	NR_043589.1	99.3%	2.990
OTU 0164	*Porphyromonas asaccharolytica* DSM 20707	NR_074588.1	100.0%	2.802
	Porphyromonas uenonis JCM 13868	NR_113091.1	98.2%	
OTU 0196	*Solobacterium moorei* JCM 10645	NR_113039.1	100.0%	2.360
OTU 0222	*Gemella morbillorum* 2917B	NR_025904.1	100.0%	2.552
OTU 0235	*Peptostreptococcus anaerobius* NCTC 11460	NR_042847.1	99.6%	2.439
OTU 0306	*Porphyromonas gingivalis* DSM 20709	NR_119038.1	100.0%	2.316
OTU 0335	*Alloprevotella tannerae* VPI N14B	NR_037088.1	98.6%	2.353
OTU 0399	*Dialister pneumosintes* ATCC 33048	NR_026229.1	100.0%	2.072
OTU 0410	*Fusobacterium nucleatum* subsp. *animalis* JCM 11025	NR_113378.1	99.6%	2.147
OTU 0456	*Fusobacterium nucleatum* subsp. *vincentii* ATCC 49256	NR_117840.1	98.2%	2.262
	Fusobacterium nulceatum subsp. *fusiforme* ATCC 51190	NR_117841.1	97.8%	
	Fusobacterium nulceatum subsp. *nucleatum* ATCC 25586	NR_074412.1	97.4%	

Supplementary Fig. 1. **Workflow charts Clinical information of Cohort-1.**

a, Workflow charts for enrolling Healthy individuals (HI) and CRC patients for microbiome analysis in Cohort-1. **b,** Distributions of age, body-mass index (BMI), sex, habit of alcohol drinking and smoking, personal history of lifestyle diseases and diabetes in analysed subjects (384 HI and 380 CRC patients). The boxes in the graph of age and BMI represent 25th-75th percentiles, black lines indicate the median, whiskers extend to the maximum and minimum values within 1.5× the interquartile range and dots indicate outliers. Statistical significance was determined with a two-tailed Wilcoxon rank-sum test (age and BMI) or a Fisher's exact test (sex, alcohol drinking, smoking, lifestyle diseases and diabetes). P values <0.05 were considered significant. Source data are provided as a source data file. **c,** Tumour profiles (depth of tumour invasion, cancer stage and tumour location) of CRC patients subjected to analysis according to the Third English Edition of the Japanese Classification of Colorectal, Appendiceal, and Anal Carcinoma. Tis or T1 cancer is defined as early CRC and T2 or deeper cancer is defined as advanced CRC. Right-sided colon: the colon from cecum to transverse colon, left-sided colon: the colon from descending colon to sigmoid colon, rectum: the rectosigmoid, the upper rectum, the lower rectum and the anal canal. **d,** Bacterial species most likely to correspond to the OTUs shown in Fig.1a. The accession numbers of the gene sequences corresponding to representative sequencing reads of each OTU are shown, along with their similarity. Linear discriminant analysis effect size (LEfSe) analysis was used to calculate the Linear Discriminant Analysis (LDA) score.

Supplementary Fig. 2

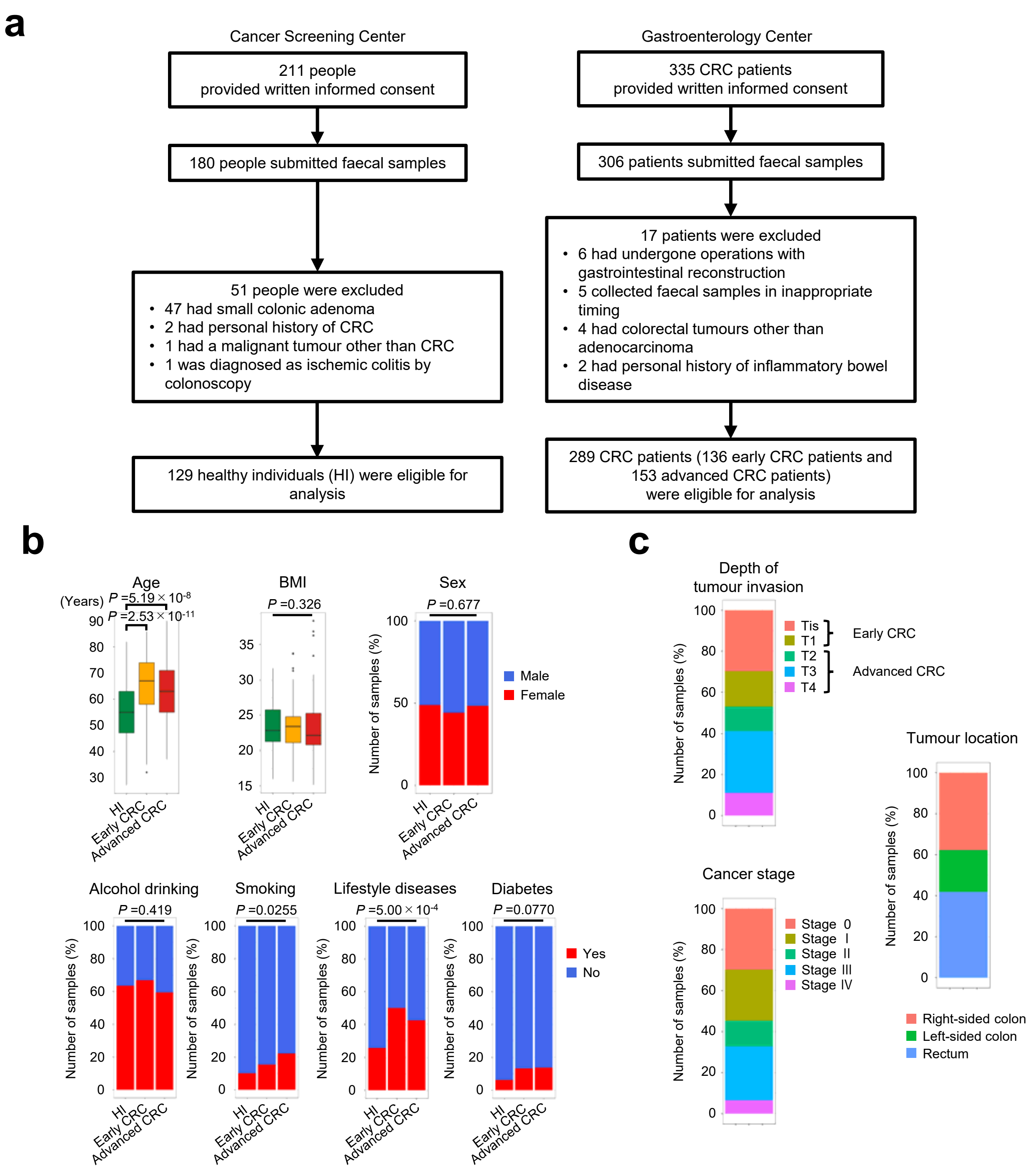

d

OTU ID	Results of the sequence similarity search using BLAST		
	Bacterial species most likely to match	Accession no.	Identity
OTU 0090	*Parvimonas micra* JCM 12970	NR_114338.1	100.0%
OTU 0273	*Prevotella intermedia* JCM 12248	NR_113106.1	99.6%
OTU 0316	*Peptostreptococcus stomatis* W2278	NR_043589.1	99.7%
OTU 0158	*Porphyromonas asaccharolytica* DSM 20707	NR_074588.1	98.5%
	Porphyromonas uenonis JCM 13868	NR_113091.1	99.6%
OTU 0224	*Solobacterium moorei* JCM 10645	NR_113039.1	100.0%
OTU 0189	*Gemella morbillorum* 2917B	NR_025904.1	100.0%
OTU 0296	*Peptostreptococcus anaerobius* NCTC 11460	NR_042847.1	99.6%
OTU 0354	*Porphyromonas gingivalis* DSM 20709	NR_119038.1	99.6%
OTU 0357	*Alloprevotella tannerae* VPI N14B	NR_037088.1	98.6%
OTU 0336	*Dialister pneumosintes* ATCC 33048	NR_026229.1	100.0%
OTU 0411	*Fusobacterium nucleatum* subsp. *animalis* JCM 11025	NR_113378.1	99.6%
OTU 0364	*Fusobacterium nucleatum* subsp. *vincentii* ATCC 49256	NR_117840.1	100.0%
	Fusobacterium nulceatum subsp. *fusiforme* ATCC 51190	NR_117841.1	99.6%
	Fusobacterium nulceatum subsp. *nucleatum* ATCC 25586	NR_074412.1	97.1%

Supplementary Fig. 2. Workflow charts and clinical information of Cohort-2. **a,** Workflow charts for enrolling Healthy individuals (HI) and CRC patients for microbiome analysis in Cohort-2. **b,** Distributions of age, BMI, sex, habit of alcohol drinking and smoking, personal history of lifestyle diseases and diabetes in analysed subjects (129 HI, 136 early CRC patients and 153 advanced CRC patients). The boxes in the graph of age and BMI represent 25th-75th percentiles, black lines indicate the median, whiskers extend to the maximum and minimum values within 1.5× the interquartile range and dots indicate outliers. Statistical significance was determined with a Kruskal-Wallis rank-sum test followed by two-tailed pairwise Wilcoxon rank sum tests (age and BMI) or a Fisher's exact test (sex, alcohol drinking, smoking, lifestyle diseases and diabetes). *P* values <0.05 were considered significant. Source data are provided as a source data file. **c,** Tumour profiles (depth of tumour invasion, cancer stage and tumour location) of CRC patients subjected to analysis. Tis or T1 cancer is defined as early CRC and T2 or deeper cancer is defined as advanced CRC. **d,** Bacterial species most likely to correspond to OTUs shown in Fig.1b were identified judging from a combination of SILVA database analysis and BLAST analysis using the V1-V2 region of the bacterial 16S rRNA gene sequence. The accession numbers of the gene sequences corresponding to representative sequencing reads of each OTU are shown, along with their similarity.

Supplementary Fig. 3

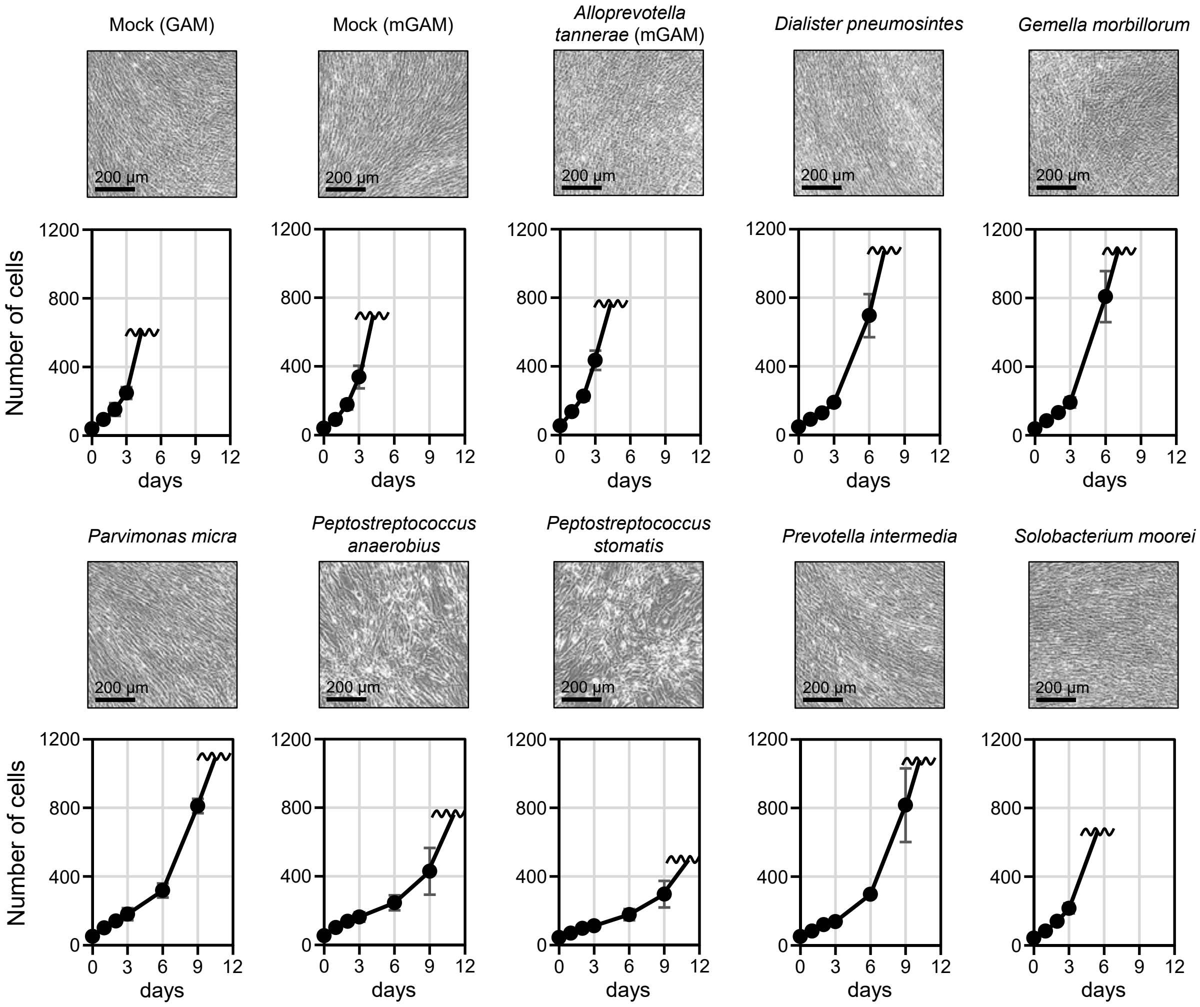

Supplementary Fig. 3 The effects of bacterial conditioned medium on cell proliferation.

Early passage TIG-3 cells were cultured with tissue culture media containing indicated bacterial conditioned media or plain bacterial culture media (Mock) at the ratio of 1/30 for 9 days, changing the medium every 3 days. These cells were then subsequently cultured with plain tissue culture media for another 3 days. Cell number was counted throughout the experiments and representative photographs of the cells in the indicated culture conditions on day 12 are shown at the top of the panels. mGAM was used as a bacterial culture media for *Alloprevotella tannerae* and GAM was used for other bacteria. For all graphs, error bars indicate mean ± standard deviation (s.d.) with three biologically independent replicates.

Supplementary Fig. 4

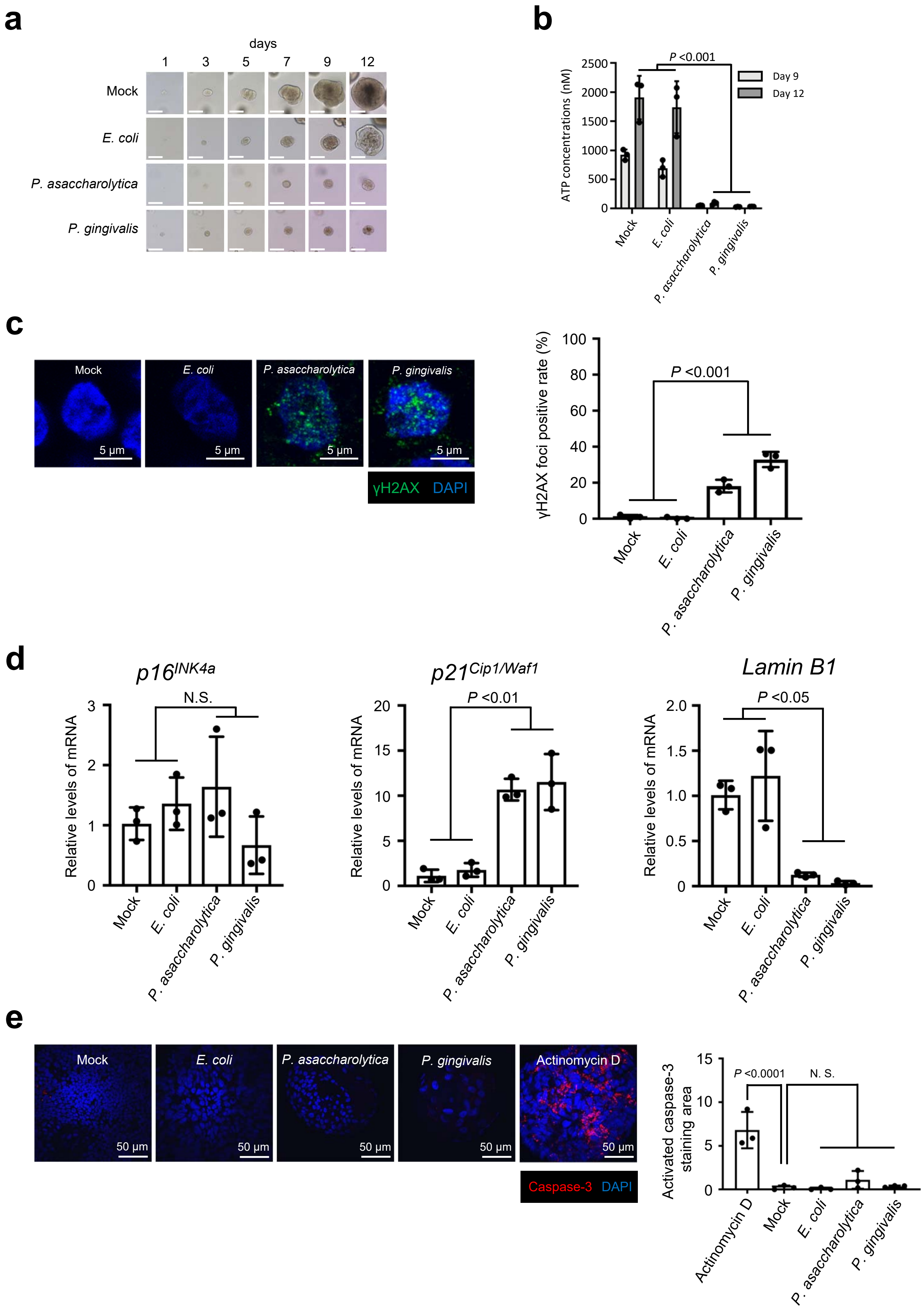

Supplementary Fig. 4 The effects of bacterial conditioned medium on cell proliferation in human intestinal organoids. Intestinal organoids differentiated from human iPSC were cultured with organoid culture media containing indicated bacterial conditioned media or plain bacterial culture media (Mock) at the ratio of 1/30 for 9 days, changing the medium every 2 or 3 days. These cells were then subsequently cultured with plain organoid culture media for another 3 days (**a** and **b**), or to subjected to immunofluorescence staining for markers of DNA damage (γ-H2AX (green) and DNA staining with 4, 6-diamidino-2-phenylindole (DAPI) (blue) (**c**), RT-qPCR analysis for indicated genes (**d**), or to the analysis for the detection of activated caspase 3 (red) positive cells (apoptotic cells) (**e**) . Representative photographs of organoids cultured under each culture condition are shown. Scale bars represent 200 µm. (**a**), 5 µm. (**c**), 50 µm. (**e**). Relative number of cells in organoids cultured under each condition on day 9 and 12 was determined by measuring the amount of ATP (**b**). The histograms indicate the percentage of nuclei that contain more than 3 foci positive for γ-H2AX staining (**c**) or the percentages of activated caspase 3 staining area (**e**) . These assays were performed in triplicate (both biological and technical replicates) and representative data were shown (**a**). For all bar graphs, error bars indicate mean ± s.d. with three biologically independent replicates (**b to e**). The conditioned media from *E. coli* culture were used as a negative control. Statistical significance was determined with one-way ANOVA followed by Tukey's test (**b**), (**c**), (**d**) or two-tailed Dunnett's test for comparing with mock (**e**). P values <0.05 were considered significant. Source data are provided as a source data file.

Supplementary Fig. 5

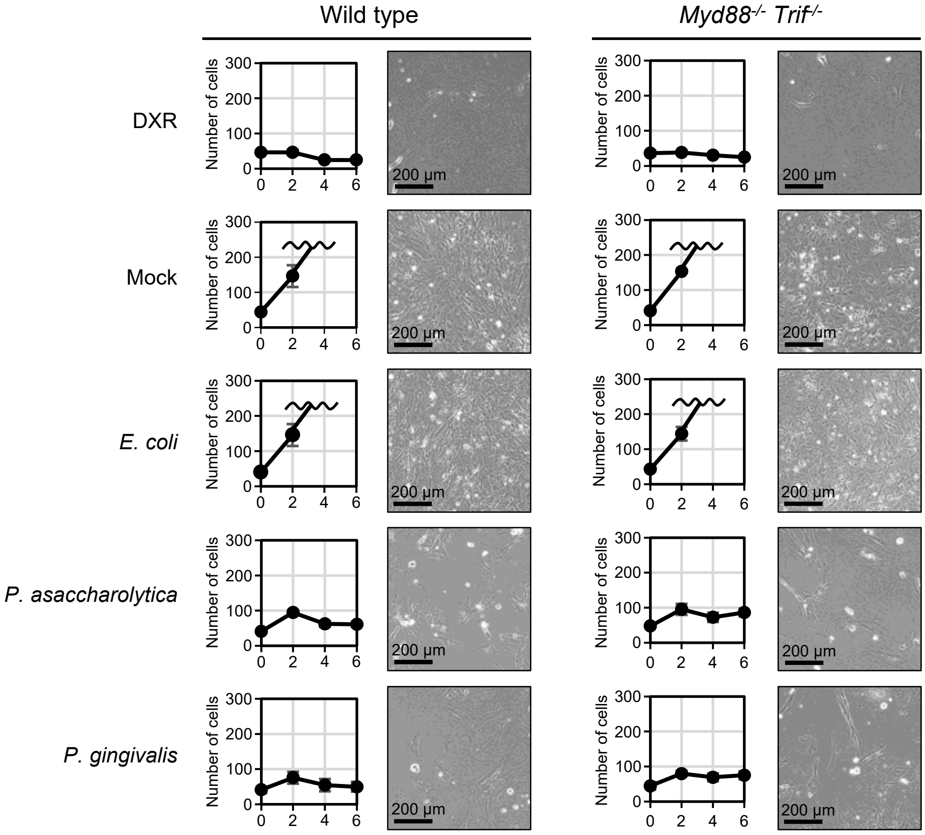

Supplementary Fig. 5. The effects of bacterial conditioned medium on cell proliferation of MEFs.

Early passage mouse embryonic fibroblasts (MEFs) of indicated genotypes were cultured with tissue culture media containing indicated bacterial conditioned media or plain bacterial culture media supplemented with (DXR) or without (Mock) 200 ng/ml doxorubicin at the ratio of 1/30 for 6 days. Cell number was counted throughout the experiments and representative photographs of the cells in the indicated culture conditions on day 6 are shown right. The conditioned media from *E. coli* culture were used as a negative control and DXR was used as a positive control. For all graphs, error bars indicate mean ± s.d. with three biologically independent replicates.

Supplementary Fig. 6

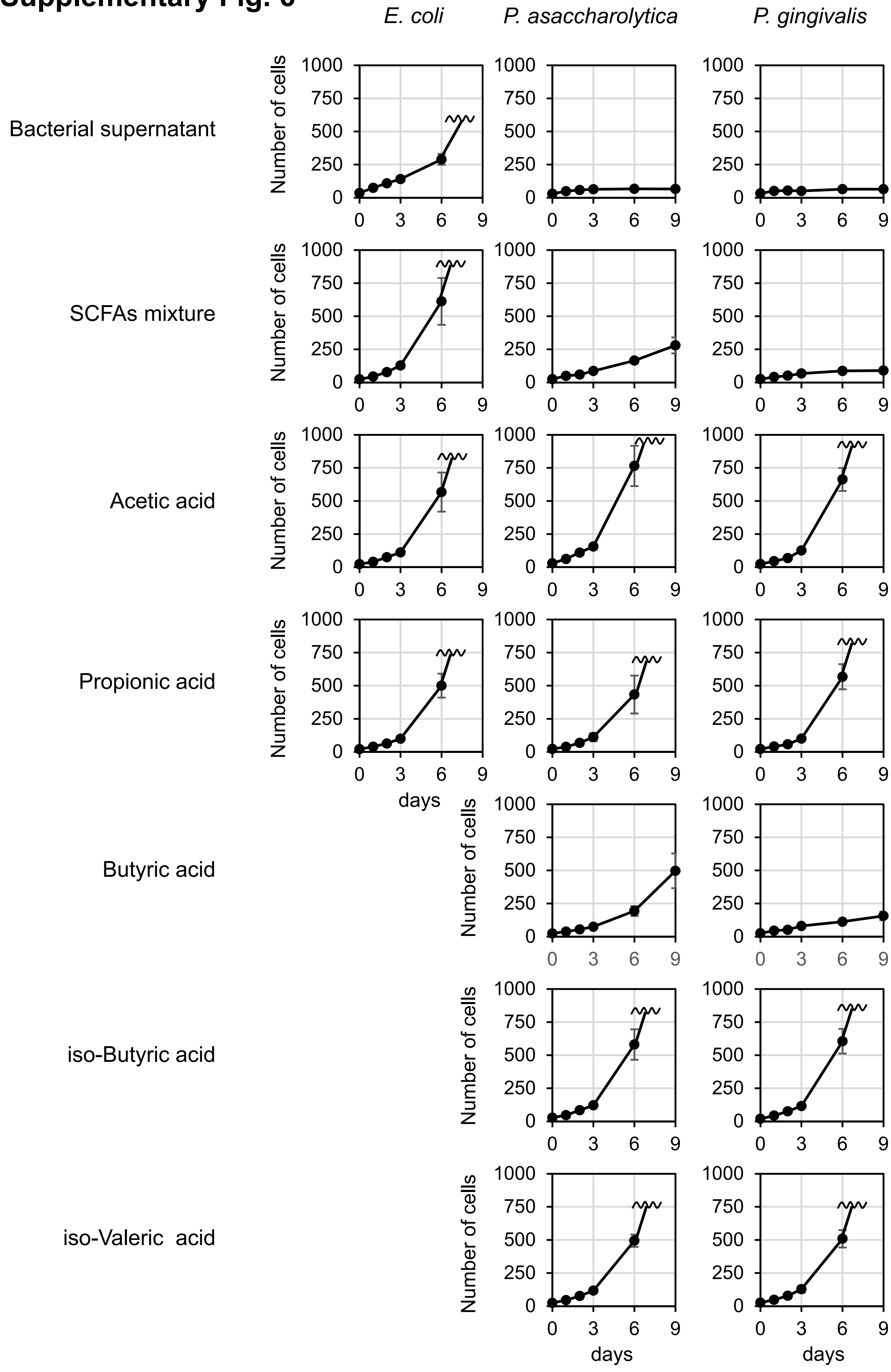

Supplementary Fig. 6. The effects of SCFAs on cell proliferation of HDFs.

Early passage TIG-3 cells were cultured for 9 days with tissue culture media containing indicated bacterial conditioned media at the ratio of 1/30 or indicated each SCFA or the mixture of these SCFAs in the same concentrations present in the bacterial conditioned media for 9 days, changing the medium every 3 days. Cell numbers were counted throughout the experiments, and representative photographs of the cells in the indicated culture conditions on day 9 are shown in Fig. 3b. These assays were performed in triplicate (both biological and technical replicates) and representative data were shown. For all graphs, error bars indicate mean ± s.d. with three biologically independent replicates.

Supplementary Fig. 7

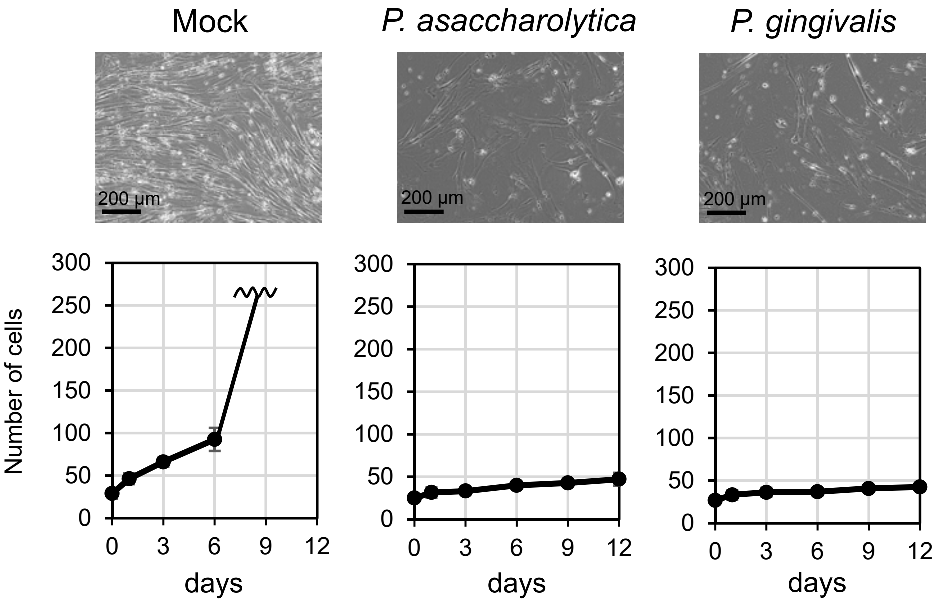

Supplementary Fig. 7 Effects of butyrate on cell proliferation in normal human colonic epithelial cells.

Early passage CCD 841 CoN cells were cultured with tissue culture media with or without (Mock) the mixture of short-chain fatty acids in the same concentrations present in the indicated bacterial conditioned media for 9 days, changing the medium every 3 days, and then subsequently cultured with plain tissue culture medium for another 3 days. Cell numbers were counted throughout the experiments, and representative photographs of the cells in the indicated culture conditions on day 12 are shown at the top of the panels. These assays were performed in triplicate (both biological and technical replicates) and representative data were shown. For all graphs, error bars indicate mean ± s.d. with three biologically independent replicates. The conditioned media from *E. coli* culture were used as a negative control. Source data are provided as a source data file.

Supplementary Fig. 8

a

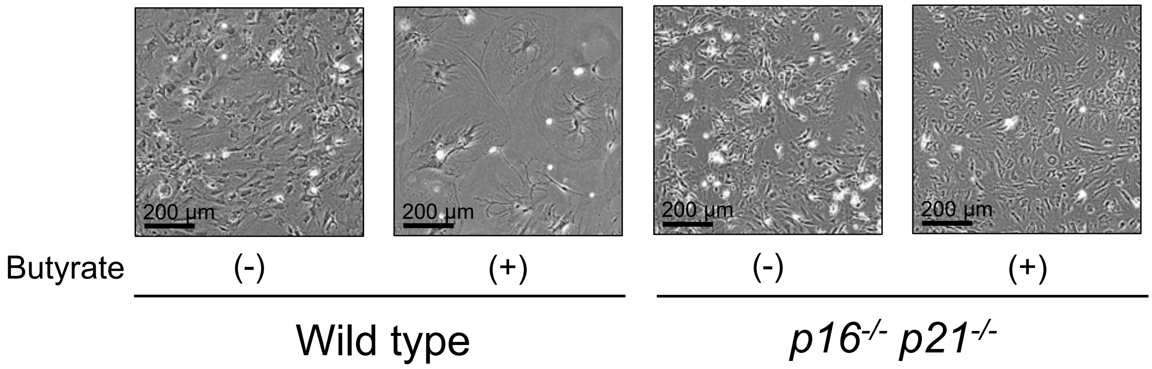

b

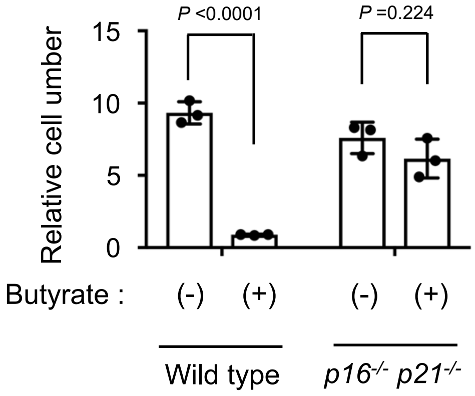

c

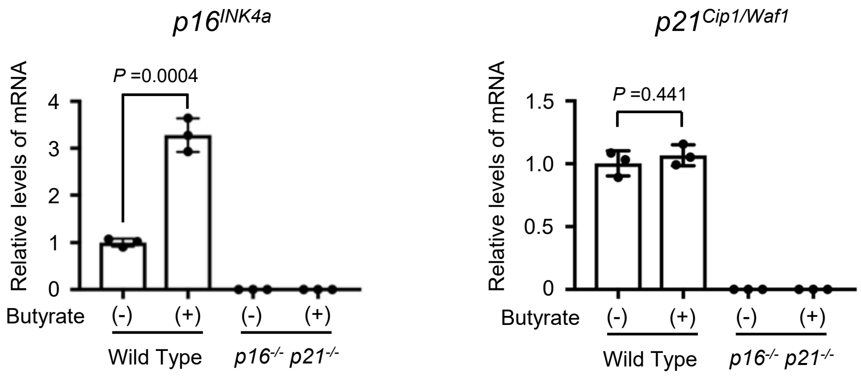

Supplementary Fig. 8. The effect of butyrate on cells lacking *p16*^{INK4a} and *p21*$^{Cip1/Waf1}$ genes.

The same number of early passage mouse skin fibroblasts prepared from the indicated genotypes were cultured in tissue culture medium with or without 5 mM concentrations of sodium butyrate for 9 days, and then subsequently cultured with plain tissue culture medium for another 8 days. These assays were performed in triplicate (both biological and technical replicates) and representative photographs of cells on day 17 cultured under the conditions indicated are shown (**a**). Cell counts were made on day day 17 (**b**). Cells were then subjected to RT-qPCR analysis for the expression of *p16*^{INK4a} and *p21*$^{Waf1/Cip1}$ genes (**c**). For all bar graphs, error bars indicate mean ± s.d. with three biologically independent replicates. Statistical significance was determined with a two-tailed Student's *t*-test. *P* values <0.05 were considered significant. Source data are provided as a source data file.

Supplementary Fig. 9

a

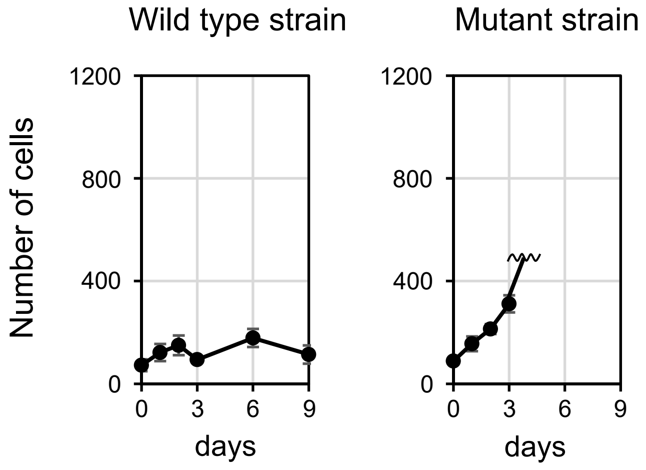

b

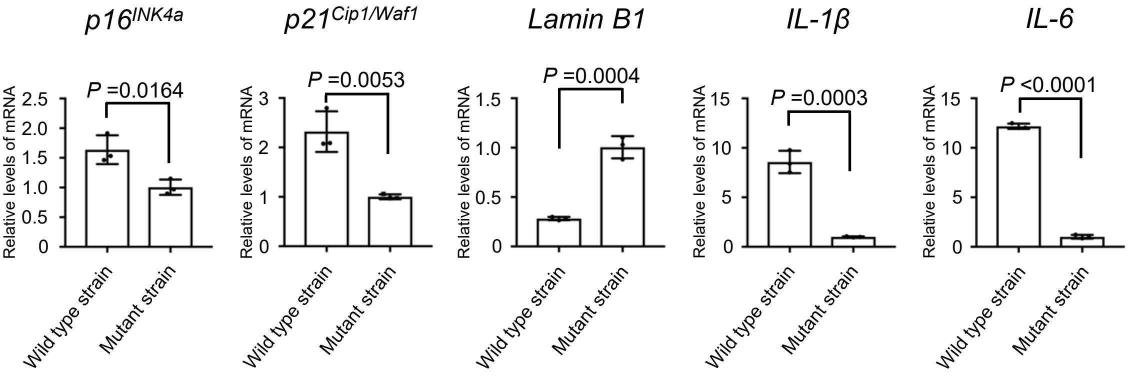

Supplementary Fig. 9. Effects of butyrate synthesis mutant of *P. gingivalis* on cell proliferation in HDFs.

Early passage TIG-3 cells were with tissue culture media containing the indicated bacterial conditioned media at a ratio of 1/30 for 9 days, changing the medium every 3 days. Cell numbers were counted throughout the experiments (**a**), and representative photographs of the cells in the indicated culture conditions on day 9 are shown in Fig. 3f. Cells on day 9 were subjected to RT-qPCR analysis for indicated genes (**b**). For all graphs, error bars indicate mean ± s.d. with three biologically independent replicates. The representative data from three biologically independent experiments were shown. These assays were performed in triplicate (both biological and technical replicates) and representative data were shown. Statistical significance was determined with a two-tailed Student's *t*-test. *P* values <0.05 were considered significant. Source data are provided as a source data file.

Supplementary Fig. 10

a

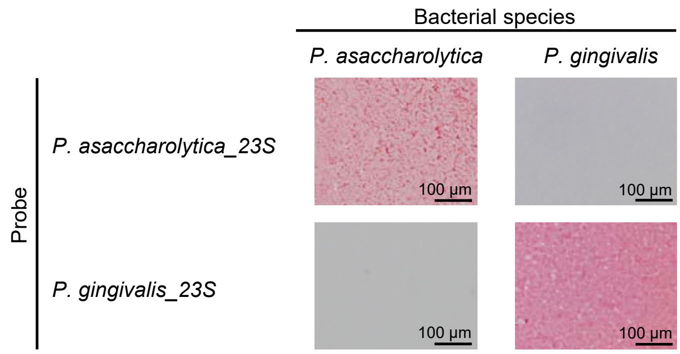

b

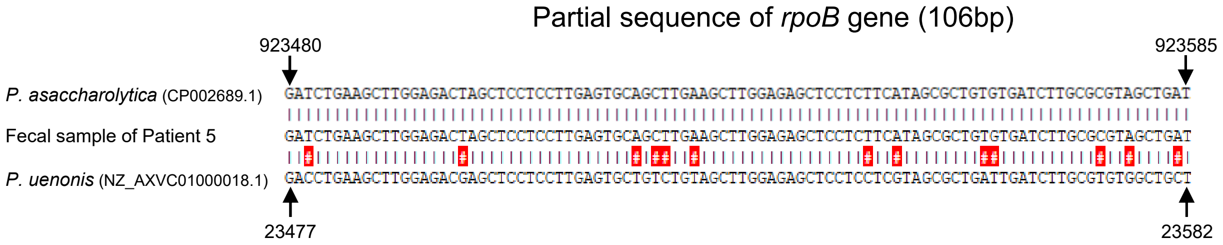

c

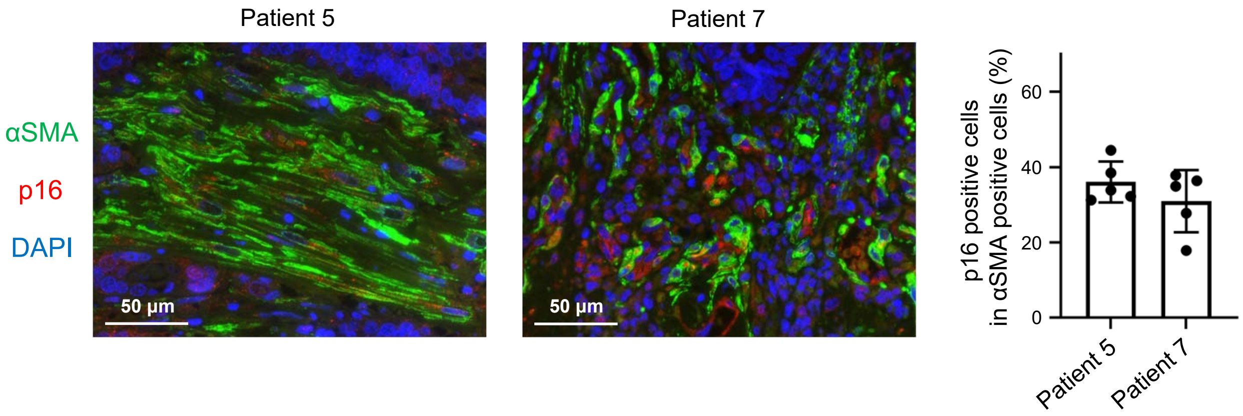

Supplementary Fig. 10. The specificity of *in situ* hybridization probes.

a, *In situ* hybridization probes for *P. asaccharolytica* and *P. gingivalis* were designed by targeting 23S rRNA genes of each bacterial species. The specificity of each probe was confirmed by cross-hybridization between the paraffin-embedded bacteria listed in the upper part of the panel and the probes listed in the left part of the panel. **b,** *rpoB* was isolated from the patient 5 and sequenced to distinguish between *Porphyromonas asaccharolytica* and *Porphyromonas uenonis.* These assays were performed over three biological independent replicates and representative data were shown (**a and b**). **c,** Immunofluorescence analysis of patient's CRC tissues using antibodies against αSMA (green) and $p16^{INK4a}$ (red). DNA was stained by 4, 6-diamidino-2-phenylindole (DAPI) (blue). These assays were performed with technical duplicates and representative data were shown. Cell count was performed in five high-power fields of each sample. The histogram indicates the percentage of αSMA-expressing cells that were positive for $p16^{INK4a}$. Error bars indicate mean ± s.d. Source data are provided as a source data file.

Supplementary Fig. 11

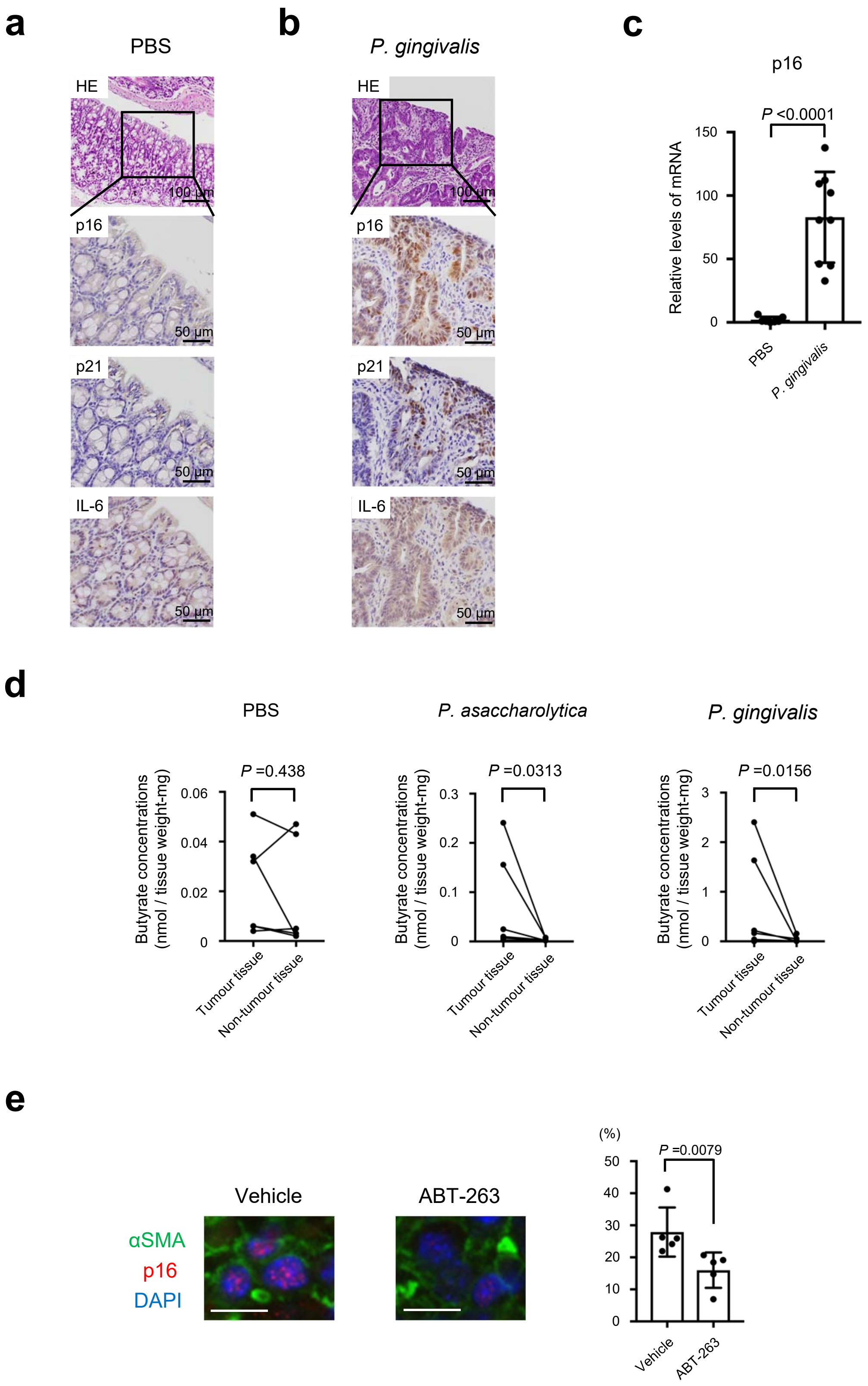

Supplementary Fig. 11 Histopathology of colonic tumours induced by P. *gingivalis*.

a and b, Paraffin-embedded tissues of normal colon or colorectal tumour of *Apc*$^{\Delta 14/+}$ mice with oral gavage of PBS **(a)** or *P. gingivalis* **(b),** respectively, were subjected to HE staining and immunostaining analysis using indicated antibodies. These assays were performed over three biologically independent animals in each group with over three technical replicate and representative images were shown. **c,** These tissues were subjected to RT-qPCR analysis for indicated genes**.** Error bars indicate mean ± s.d. with three biologically independent replicates. The representative data from three biologically independent experiments were shown. Statistical significance was determined with a two-tailed Student's *t*-test. *P* values <0.05 were considered significant. **d,** Butyrate concentrations in colorectal tumour tissues and paired non-tumour tissues of *Apc*$^{\Delta 14/+}$ mice with oral gavage of PBS or indicated bacteria. Statistical significance was determined with a two-tailed Wilcoxon signed-rank test. *P* value <0.05 was considered significant. **e,** Immunofluorescence analysis of CRC tissues of mice described in Fig. 5f using antibodies against αSMA (green) and p16^{INK4a} (red). DNA was stained by 4, 6-diamidino-2-phenylindole (DAPI) (blue). These assays were performed for five biologically independent animals in each group with technical duplicate and representative images were shown. Scale bars represent 10 μm. The histogram indicates the percentage of αSMA-expressing cells that were positive for p16^{INK4a} per mouse. Error bars indicate mean ± s.d. Statistical significance was determined with a two-tailed Wilcoxon rank-sum test. *P* value <0.05 was considered significant. Source data are provided as a source data file.
