# Supplementary material for: Gut bacteria identified in colorectal cancer patients promote tumourigenesis via butyrate secretion
Source: Nat Commun. 2021 Sep 28;12:5674. doi: 10.1038/s41467-021-25965-x (PMC8479117; doi:10.1038/s41467-021-25965-x)
Supplement: Supplementary file 2 — Reporting summary [file 41467_2021_25965_MOESM2_ESM.pdf]

nature portfolio

Corresponding author(s): Eiji Hara

Last updated by author(s): Aug 7, 2021

Reporting Summary

Nature Portfolio wishes to improve the reproducibility of the work that we publish. This form provides structure for consistency and transparency in reporting. For further information on Nature Portfolio policies, see our Editorial Policies and the Editorial Policy Checklist.

Statistics

For all statistical analyses, confirm that the following items are present in the figure legend, table legend, main text, or Methods section.

n/a	Confirmed	
☐	☒	The exact sample size (n) for each experimental group/condition, given as a discrete number and unit of measurement
☐	☒	A statement on whether measurements were taken from distinct samples or whether the same sample was measured repeatedly
☐	☒	The statistical test(s) used AND whether they are one- or two-sided<br>*Only common tests should be described solely by name; describe more complex techniques in the Methods section.*
☒	☐	A description of all covariates tested
☐	☒	A description of any assumptions or corrections, such as tests of normality and adjustment for multiple comparisons
☐	☒	A full description of the statistical parameters including central tendency (e.g. means) or other basic estimates (e.g. regression coefficient) AND variation (e.g. standard deviation) or associated estimates of uncertainty (e.g. confidence intervals)
☐	☒	For null hypothesis testing, the test statistic (e.g. F, t, r) with confidence intervals, effect sizes, degrees of freedom and P value noted<br>*Give P values as exact values whenever suitable.*
☒	☐	For Bayesian analysis, information on the choice of priors and Markov chain Monte Carlo settings
☒	☐	For hierarchical and complex designs, identification of the appropriate level for tests and full reporting of outcomes
☒	☐	Estimates of effect sizes (e.g. Cohen's d, Pearson's r), indicating how they were calculated

Our web collection on statistics for biologists contains articles on many of the points above.

Software and code

Policy information about availability of computer code

Data collection	Meta-16S rRNA gene sequencing data: Illumina Miseq (Illumina)<br>Microscopic data: Fluorescence Microscope BZ-X710 (KEYENCE), FLUOVIEW FV3000 Confocal Laser Scanning Microscope (OLYMPUS)<br>Quantitative real-time PCR data: StepOnePlus PCR system (Applied Biosystems)<br>Immunoblotting data: LAS-3000mini imaging system (Fujifilm)<br>ROS measurement data: ARVO MX/Light 1420 Multilabel/Luminescence Counter (PerkinElmer)<br>RNA sequencing data: Illumina HiSeq 2500 platform (Illumina)<br>Histological data: BX53 Upright Microscope (OLYMPUS), Fluorescence Microscope BZ-X710 (KEYENCE)<br>DNA sequencing data: Applied Biosystems 3500xL Genetic Analyzer (Thermo Fisher Scientific)<br>SCFA concentration data: LC-10ADvp (Shimadzu), Nexera UHPLC (Shimadzu)
Data analysis	16S rRNA gene sequencing analysis: QIIME2 (2019.10.0), LEfSe (1.0.8.post1), BLAST (2.9.0)<br>RNA sequencing analysis: STAR (2.7.4a), FastQC (0.11.9), HTSeq (0.12.4), edgeR (version 3.30.3)<br>Immunoblotting analysis: Multi Guage V 3.1 (Fujifilm)<br>Immunofluorescence staining analysis: Image J 1.53e<br>Histological data: cellSens Standard 2.1 (OLYMPUS)<br>DNA sequencing data: A plasmid Editor version 2<br>Statistical analysis: GraphPad Prism 9, R (version 4.0.2)

For manuscripts utilizing custom algorithms or software that are central to the research but not yet described in published literature, software must be made available to editors and reviewers. We strongly encourage code deposition in a community repository (e.g. GitHub). See the Nature Portfolio guidelines for submitting code & software for further information.

Data

Policy information about availability of data

All manuscripts must include a data availability statement. This statement should provide the following information, where applicable:

- Accession codes, unique identifiers, or web links for publicly available datasets
- A description of any restrictions on data availability
- For clinical datasets or third party data, please ensure that the statement adheres to our policy

Microbiome analysis (bacterial 16S rRNA gene meta-sequence) data and RNA sequencing (TIG-3 cells) data that support the findings of this study have been deposited in the DNA Data Bank of Japan (DDBJ) with the accession codes DRA011735 or DRA011736, respectively (https://www.ddbj.nig.ac.jp). The deposited data are available in NCBI under accession number DRA011735 (https://www.ncbi.nlm.nih.gov/sra/?term=DRA011735), DRA011736 (https://www.ncbi.nlm.nih.gov/sra/?term=DRA011736). The databases referred in microbiome analysis are as follows: SILVA (https://www.arb-silva.de/) and National Center for Biotechnology Information (NCBI) (https://www.ncbi.nlm.nih.gov/). Bacterial gene data mentioned in the text are available in NCBI under accession number NC_015501.1 (https://www.ncbi.nlm.nih.gov/nuccore/NC_015501.1/), NC_00729.1 (https://www.ncbi.nlm.nih.gov/nuccore/NC_010729.1/), CP002689.1 (https://www.ncbi.nlm.nih.gov/nuccore/CP002689.1), NZ_BAJM01000009 (https://www.ncbi.nlm.nih.gov/nuccore/NZ_BAJM01000009), PGN_0725 (https://www.ncbi.nlm.nih.gov/gene/?term=PGN_0725), PGN_1341 (https://www.ncbi.nlm.nih.gov/gene/?term=PGN_1341), and PGN_1888 (https://www.ncbi.nlm.nih.gov/gene/?term=PGN_1888).
The database referred in RNA sequencing analysis is available in Ensembl (https://www.ensembl.org/).
The source data underlying Figs 2b-f, 3b, 3c, 4a, 4c, 4f, 5c-f and Supplementary Figs 3, 4b-e, 5, 6, 7, 8b, 8c, 9, 10c, 11c-e are provided as a Source Data File. Uncropped and unprocessed scans of the blots are included in the Source Data file. The remaining data are available from the authors upon reasonable request.

Field-specific reporting

Please select the one below that is the best fit for your research. If you are not sure, read the appropriate sections before making your selection.

[x] Life sciences [ ] Behavioural & social sciences [ ] Ecological, evolutionary & environmental sciences

For a reference copy of the document with all sections, see nature.com/documents/nr-reporting-summary-flat.pdf

Life sciences study design

All studies must disclose on these points even when the disclosure is negative.

Sample size	For human gut microbiome analysis, no sample size calculation was performed because this study is observational, not interventional. For the other experiments, the sample size was determined based on the expense of data collection, and the need to have sufficient statistical power.
Data exclusions	For human gut microbiome analysis, faecal samples of participants matching to exclusion criteria were excluded before subjected to meta-16S rRNA sequencing. Participants having clinical characteristics that could affect gut microbiome or collecting faecal samples in inappropriate timing were excluded according to the exclusion criteria. For the other experiments, no data was excluded from the analysis.
Replication	All data presented were obtained from three or two independent experiments with similar outcomes. (see Figure legends)
Randomization	For human gut microbiome analysis, randomization was not applicable because this was an observational study. In animal experiments, mice were randomized to receive treatments.
Blinding	For the analysis of meta-16S rRNA gene sequencing, blinding was not possible because statistical tests for difference between groups depended on clinical profiles. For other experiments, data collection and analysis were not performed blind. Controls and relative group samples were processed either simultaneously or in parallel.

Reporting for specific materials, systems and methods

We require information from authors about some types of materials, experimental systems and methods used in many studies. Here, indicate whether each material, system or method listed is relevant to your study. If you are not sure if a list item applies to your research, read the appropriate section before selecting a response.

Materials & experimental systems

n/a	Involved in the study	
[ ]	[x]	Antibodies
[ ]	[x]	Eukaryotic cell lines
[x]	[ ]	Palaeontology and archaeology
[ ]	[x]	Animals and other organisms
[ ]	[x]	Human research participants
[x]	[ ]	Clinical data
[x]	[ ]	Dual use research of concern

Methods

n/a	Involved in the study	
[x]	[ ]	ChIP-seq
[x]	[ ]	Flow cytometry
[x]	[ ]	MRI-based neuroimaging

Antibodies

Antibodies used	γH2AX (Millpore, cat#: 05-636, clone JBW301, lot#: 3153259), phospho-(Ser/Thr) ATM/ATR substrate (Cell Signaling Technology, cat#: 2851, lot#: 8), Cleaved Caspase-3 (Asp175) (Cell Signaling Technology, cat#: 9661, lot#: 43), p16INK4a (IBL, cat#: 11104, clone 1H4, lot#: 1L309 or Abgent, cat#: ALS16384, clone 5A8A4,3G8D12, lot#: 74347 or Abcam, cat#: ab211542, clone EPR20418, lot#: GR3232279-7 or Abcam, cat#: ab81278, clone EP435Y-129R, lot#: GR106921-28), p21Cip1/Waf1 (Cell Signaling Technology, cat#: 2947, clone 12D1, lot#: 11 or Abcam, cat#:ab107099, clone HUGO291, lot#: GR293812-6), LaminB1 (Abcam, cat#: ab16048, lot#: GR3223716-1), RB (Santa Cruz, cat#: sc-102, lot#: D1318), phospho-RB (Ser780) (Cell Signaling Technology, cat#: 9307, lot#: 13), p53 (Santa Cruz, cat#: sc-6243, lot#: K0165), phospho-p53 (Ser15) (Cell Signaling Technology, cat#: 9284, lot#: 21), α-tubulin (Sigma Aldrich, cat#: T9026, clone DM1A, lot#: 047M4789V), IL-6 (Abcam, cat#: ab6672, lot#: GR3195128-4), α-SMA (Sigma-Aldrich, cat#: A5228, clone 1A4, lot#: 074M4814V, Abcam, ab125057, clone 1A4, lot#: GR276535-1), Anti-mouse IgG (Cell Signaling Technology, cat#: 7076, lot#: 32), Anti-rabbit IgG (Cell Signaling Technology, cat#: 7074, lot#: 26), Goat anti-rabbit IgG (Vector Laboratories, cat#: BA-1000, lot#: ZF0809), Goat anti-rat IgG (Vector Laboratories, cat#: BA-9400, lot#: ZB1216), Donkey anti-mouse IgG Alexa Fluor 488 (Thermo Fisher Scientific, cat#: A21202, lot#: 1696430), Donkey anti-rabbit IgG Alexa Fluor 594 (Thermo Fisher Scientific, cat#: A21207, lot#: 1668652), Donkey anti-rabbit IgG Alexa Fluor Plus 555 (Thermo Fisher Scientific, cat#: A32794, lot#: UI284002)
Validation	All antibodies used in this study were commercially available antibodies and were validated by companies. Data sheets are available from the web links as described below.<br>γH2AX; https://www.merckmillipore.com/JP/ja/product/Anti-phospho-Histone-H2A.X-Ser139-Antibody-clone-JBW301,MM_NF-05-636-I<br>phospho-(Ser/Thr), ATM/ATR substrate; https://www.cellsignal.com/products/primary-antibodies/phospho-ser-thr-atm-atr-substrate-antibody/2851<br>Cleaved Caspase-3; https://www.cellsignal.jp/products/primary-antibodies/cleaved-caspase-3-asp175-antibody/9661<br>p16INK4a; https://www.ibl-america.com/p16-ink4a-1h4-anti-human-mouse-igg-moab/<br>p16INK4a; http://www.abcepta.com/products/ALS16384-Anti-CDKN2A--p16INK4a-Antibody-clone-5A8A43G8D12-ALS16384<br>p16INK4a; https://www.abcam.com/cdkn2ap16ink4a-antibody-epr20418-ab211542.html<br>p16INK4a; https://www.abcam.co.jp/cdkn2ap16ink4a-antibody-ep435y-129r-ab81278.html<br>p21Cip1/Waf1; https://www.cellsignal.jp/products/primary-antibodies/p21-waf1-cip1-12d1-rabbit-mab/2947<br>p21Cip1/Waf1; https://www.abcam.co.jp/p21-antibody-HUGO291-ab107099.html<br>LaminB1; https://www.abcam.co.jp/lamin-b1-antibody-nuclear-envelope-marker-ab16048.html<br>RB; https://www.scbt.com/p/rb-antibody-if8/<br>phospho-RB; https://www.cellsignal.com/products/primary-antibodies/phospho-rb-ser780-antibody/9307<br>p53; https://www.scbt.com/p/p53-antibody-fl-393<br>phospho-p53; https://www.cellsignal.com/products/primary-antibodies/phospho-p53-ser15-antibody/9284<br>α-tubulin; https://www.sigmaaldrich.com/catalog/product/sigma/t9026<br>IL-6; https://www.abcam.co.jp/il-6-antibody-ab6672.html<br>α-SMA; https://www.sigmaaldrich.com/catalog/product/SIGMA/A5228<br>α-SMA; https://www.abcam.co.jp/biotin-alpha-smooth-muscle-actin-antibody-1a4-ab125057.html<br>Anti-mouse IgG; https://www.cellsignal.jp/products/secondary-antibodies/anti-mouse-igg-hrp-linked-antibody/7076<br>Anti-rabbit IgG; https://www.cellsignal.jp/products/secondary-antibodies/anti-rabbit-igg-hrp-linked-antibody/7074<br>Goat anti-rabbit IgG; https://vectorlabs.com/biotinylated-goat-anti-rabbit-igg-antibody.html<br>Goat anti-rat IgG; https://vectorlabs.com/catalogsearch/result/?q=BA-9400<br>Donkey anti-mouse IgG Alexa Fluor 488; https://www.thermofisher.com/antibody/product/Donkey-anti-Mouse-IgG-H-L-Highly-Cross-Adsorbed-Secondary-Antibody-Polyclonal/A-21202<br>Donkey anti-rabbit IgG Alexa Fluor 594; https://www.thermofisher.com/antibody/product/Donkey-anti-Rabbit-IgG-H-L-Highly-Cross-Adsorbed-Secondary-Antibody-Polyclonal/A-21207<br>Donkey anti-rabbit IgG Alexa Fluor Plus 555; https://www.thermofisher.com/antibody/product/Donkey-anti-Rabbit-IgG-H-L-Highly-Cross-Adsorbed-Secondary-Antibody-Polyclonal/A32794

Eukaryotic cell lines

Policy information about cell lines

Cell line source(s)	TIG-3 cells, mouse embryonic fibroblasts (MEFs) and CCD 841 CoN cells were obtained from Japanese Cancer Research Resources Bank (JCRB), Oriental BioService, ATCC respectively. The human induced pluripotent stem cell (iPSC) line TkDN4-M was supplied by the University of Tokyo. Mouse skin fibroblasts were isolated from the skin of a C57BL/6J wild type mouse and that of a C57BL/6J p16-/- p21-/- mouse.
Authentication	Authentication of TkDN4-M was described in reference article 68. Authentication of mouse skin fibroblasts was not necessary because developed in our lab. Other cells were obtained from public bioresources bank or Company and were not authenticated by ourselves.
Mycoplasma contamination	We have confirmed that there were not mycoplasma contamination in our tissue culture cells.
Commonly misidentified lines<br>(See ICLAC register)	No commonly misidentified cell lines were used in this study.

Animals and other organisms

Policy information about studies involving animals; ARRIVE guidelines recommended for reporting animal research

Laboratory animals	6-week-old 63 male and 74 female C57BL/6J ApcΔ14/+ mice were used.
Wild animals	The study did not involve wild animals.
Field-collected samples	The study did not involve samples collected from the field.
Ethics oversight	All mouse experiments were approved by the Animal Research Committee of Research Institute for Microbial Diseases, Osaka University.

Note that full information on the approval of the study protocol must also be provided in the manuscript.

Human research participants

Policy information about studies involving human research participants

Population characteristics	A total of 764 (383 males and 381 females, aged between 29 and 93 years old, 60.8 on average) individuals in Cohort-1 and 418 (217 males and 201 females, aged between 27 and 90 years old, 61.2 on average) individuals in Cohort-2 were subjected to gut microbiome analysis by meta-16S rRNA gene sequencing. (see Supplementary Fig. 1 and Supplementary Fig. 2)
Recruitment	This study was conducted with subjects visited the Cancer Institute Hospital of Japanese Foundation for Cancer Research (JFCR), Tokyo, Japan, who were explained about this study and consent to it. The recruitment was announced for all visitors in the recruitment periods to minimize selection bias. But there could be a referral bias because this study was conducted in a single institute. So the twelve bacteria detected in CRC patients may not be detected from CRC patients in other populations.
Ethics oversight	The samples and clinical information used in this study were obtained under conditions of informed consent and with approval of the ethics committee of the JFCR and Osaka University.

Note that full information on the approval of the study protocol must also be provided in the manuscript.
